# Emergence of Compensatory Mutations Reveals the Importance of Electrostatic Interactions between HIV-1 Integrase and Genomic RNA

**DOI:** 10.1128/mbio.00431-22

**Published:** 2022-08-17

**Authors:** Christian Shema Mugisha, Tung Dinh, Abhishek Kumar, Kasyap Tenneti, Jenna E. Eschbach, Keanu Davis, Robert Gifford, Mamuka Kvaratskhelia, Sebla B. Kutluay

**Affiliations:** a Department of Molecular Microbiology, Washington University School of Medicine, Saint Louis, Missouri, USA; b Division of Infectious Diseases, University of Colorado School of Medicine, Aurora, Colorado, USA; c MRC-University of Glasgow Centre for Virus Research, Bearsden, Glasgow, United Kingdom; University of Pennsylvania

**Keywords:** HIV-1, integrase, protein-RNA interactions, virion maturation, virology

## Abstract

HIV-1 integrase (IN) has a noncatalytic function in virion maturation through its binding to the viral RNA genome (gRNA). Class II IN substitutions inhibit IN-gRNA binding and result in the formation of virions with aberrant morphologies marked by mislocalization of the gRNA between the capsid lattice and the lipid envelope. These viruses are noninfectious due to a block at an early reverse transcription stage in target cells. HIV-1 IN utilizes basic residues within its C-terminal domain (CTD) to bind to the gRNA; however, the molecular nature of how these residues mediate gRNA binding and whether other regions of IN are involved remain unknown. To address this, we have isolated compensatory substitutions in the background of a class II IN mutant virus bearing R269A/K273A substitutions within the IN-CTD. We found that the nearby D256N and D270N compensatory substitutions restored the ability of IN to bind gRNA and led to the formation of mature infectious virions. Reinstating the local positive charge of the IN-CTD through individual D256R, D256K, D278R, and D279R substitutions was sufficient to specifically restore IN-gRNA binding and reverse transcription for the IN R269A/K273A as well as the IN R262A/R263A class II mutants. Structural modeling suggested that compensatory substitutions in the D256 residue created an additional interaction interface for gRNA binding, whereas other substitutions acted locally within the unstructured C-terminal tail of IN. Taken together, our findings highlight the essential role of CTD in gRNA binding and reveal the importance of pliable electrostatic interactions between the IN-CTD and the gRNA.

## INTRODUCTION

A defining feature of retroviruses is the reverse transcription of the viral RNA genome (gRNA) and integration of the resultant linear viral DNA into the host chromosome, which establishes lifelong infection. The latter process is mediated by the viral integrase (IN) enzyme, which catalyzes 3′ processing and DNA strand transfer reactions (1). The catalytic activity of HIV-1 IN has been successfully targeted by several integrase strand-transfer inhibitors (INSTIs) (2–7) that have become key components of frontline antiretroviral therapy regimens due to their high efficacy and tolerance profiles (8–11). In addition, HIV-1 IN has a noncatalytic function in virus replication (12–16). Successful targeting of this second function can complement the existing antiviral regimens and substantially increase the barrier to INSTI resistance.

HIV-1 IN consists of three independently folded protein domains: the N-terminal domain (NTD) bears the conserved His and Cys residues (HHCC motif) that coordinate Zn^2+^ binding for 3-helix bundle formation; the catalytic core domain (CCD) adopts an RNase H fold and harbors the enzyme active site composed of an invariant DDE motif, and the C-terminal domain (CTD), which adopts an SH3 fold (17, 18). Integration is facilitated by a cellular cofactor, lens epithelium-derived growth factor (LEDGF/p75), which binds tightly to a site within the CCD dimer interface (19, 20) and guides the preintegration complex to actively transcribed regions of the host chromosome (19–24). A group of pleotropic IN mutations distributed throughout IN, collectively known as class II mutations, disrupt viral assembly (13, 16, 25–36), morphogenesis (12, 16, 27, 32–34, 37–39), and reverse transcription in target cells (12–14, 16, 29, 31, 32, 34, 36–55), often without obstructing the catalytic activity of IN *in vitro* (13, 27, 28, 31, 41, 42, 45, 47, 56–58). A hallmark of class II IN mutant viruses is the mislocalization of the viral ribonucleoprotein complexes (vRNP) outside of the viral capsid (CA) lattice, a deformation that is commonly referred to as the eccentric morphology (12, 15, 16, 27, 32–34, 37, 38, 59–61). Although originally designed to inhibit integration through preventing the binding of IN to LEDGF/p75 (59, 62–66), allosteric integrase inhibitors (ALLINIs) potently inhibit proper particle maturation (12, 37, 62) and lead to the formation of virions that display a similar eccentric morphology observed with class II IN mutations (12).

We have previously shown that IN binds to the gRNA in mature virions to mediate proper encapsidation of the viral ribonucleoproteins (vRNPs) inside the mature CA lattice (38). IN binds with high affinity to the TAR hairpin present within the 5′ and 3’UTRs, though multiple other distinct locations on the gRNA without apparent secondary structure are also bound (38). A parameter that appears to be critical for gRNA binding is functional tetramerization of IN. Numerous class II IN mutations scattered throughout IN block IN-gRNA binding indirectly by disrupting functional oligomerization of IN (15). Similar to these class II IN mutations, ALLINIs are thought to interfere with IN-gRNA binding indirectly by inducing aberrant IN multimerization (12, 38). ALLINIs bind to a pocket within the CCD of IN (12, 61, 62, 65, 67–69) and induce the formation of open IN polymers by engaging the CTD of a nearby IN dimer (63). In contrast, mutation of basic residues within IN-CTD (i.e., R262, R263, R269, and K273) inhibits IN-gRNA binding without altering IN oligomerization in virions and *in vitro* (15, 38), suggesting that these residues directly mediate IN binding to the gRNA.

While the involvement of IN-CTD in gRNA binding has been established, how the basic residues within IN-CTD mediate recognition of the gRNA remains unknown. For example, it is possible that the positively charged Lys and Arg residues interact with the negatively charged RNA phosphate backbone in a nonspecific or semispecific manner, depending on the folding and structure of the cognate RNA element, driven by electrostatic interactions (70–72). Alternatively, these residues can mediate specific interactions with gRNA through H-bonding and van der Waals contacts with individual nucleobases (70–72). Though the structural details of IN-RNA complexes remain unknown, a recent modeling study suggested that a combination of electrostatic interactions with the phosphate backbone and specific interactions of hydrophobic residues with the bases may mediate specific recognition of the TAR element by IN (73). Furthermore, it is possible that other domains in IN also contribute to gRNA binding. For example, we have previously shown that K34A substitution within the NTD blocks gRNA binding without impacting IN oligomerization (15), suggesting its direct interaction with the gRNA.

To gain insight into the mode of IN-gRNA interactions, the noninfectious HIV-1_NL4-3 IN (R269A/K273A)_ class II IN mutant virus was serially passaged in T-cells until the acquisition of compensatory mutations. Two compensatory substitutions, D256N and D270N, sequentially emerged within the IN-CTD and allowed HIV-1_NL4-3 IN (R269A/K273A)_ to replicate with WT kinetics. Introduction of these mutations on the HIV-1_NL4-3 IN (R269A/K273A)_ virus backbone substantially enhanced IN-gRNA binding, reverse transcription, and virion infectivity, and restored proper virion morphogenesis. As the d-to-N substitutions resulted in loss of two negative charges, possibly overcoming the loss of two positive charges with the R269A/K273A substitutions, we tested whether restoring the overall local charge of IN-CTD would also restore IN-gRNA binding and virion infectivity. Indeed, the D256R as well as other nearby charge reversal substitutions including D278R and D279R enhanced or fully restored RNA binding, reverse transcription, and virion infectivity for the IN R269A/K273A mutant. We further extended these findings to another class II IN mutant, R262A/R263A, which was similarly suppressed by the D256N/K/R substitutions. Compensatory substitutions did not affect the ability of IN to multimerize *in vitro* or in virions, suggesting that they restored the RNA-binding ability of IN directly. Together, our findings highlight the essential role of IN-CTD in gRNA binding and reveal a pliable electrostatic component of IN-gRNA interactions.

## RESULTS

### Compensatory IN D256N/D270N substitutions emerge in the background of the HIV-1_NL4-3 IN (R269A/K273A)_ class II IN mutant virus.

**FIG 1:**
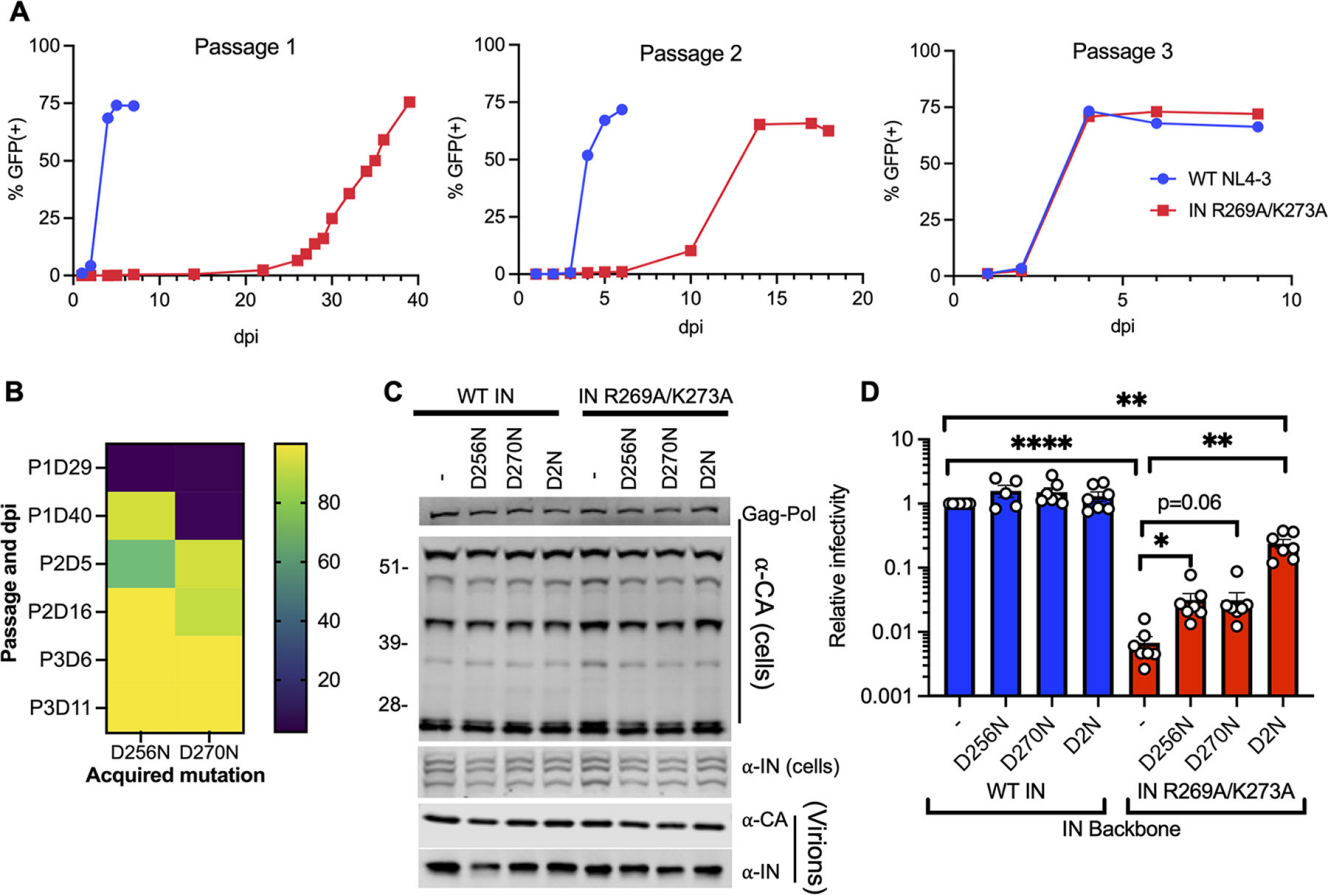
D256N and D270N compensatory substitutions in HIV-1 IN suppress the replication defect of the HIV-1_NL4-3 IN (R269A/K273A)_ class II mutant virus. (A) MT4-LTR-GFP indicator cells were infected with HIV-1_NL4-3_ at an MOI of 2 i.u./cell or an equivalent particle number of the HIV-1_NL4-3 IN (R269A/K273A)_ class II IN mutant virus (based on reverse transcriptase [RT] activity) as detailed in Materials and Methods. HIV-1_NL4-3 IN (R269A/K273A)_ viruses were serially passaged for three rounds until the emergence of compensatory mutations that allowed virus replication with WT kinetics. The graphs represent the percentage of GFP positive cells as assessed by flow cytometry over three passages at the indicated days postinfection (dpi). (B) HIV-1 genomic RNA was isolated from viruses collected from cell culture supernatants over the three passages (i.e., P1, P2, P3) and at the indicated days postinfection (i.e., D29, D40, etc.) as described in Materials and Methods. Heatmap shows the percentage of IN D256N and D270N substitutions at the indicated passages and days postinfection (dpi) as assessed by whole-genome deep sequencing. (C) HEK293T cells were transfected with full-length pNL4-3 expression plasmids carrying *pol* mutations coding for the IN D256N, D270N, and D256N/D270N (D2N) substitutions introduced on the WT IN and IN R269A/K273A backbones. Cell lysates and cell culture supernatants containing virions were collected 2 days posttransfection and analyzed by immunoblotting for CA and IN. The image is representative of five independent experiments. See Fig. S1 for quantitative analysis of immunoblots. (D) HEK293T cells were transfected as in panel C, and cell culture supernatants containing viruses were titered on TZM-bl indicator cells, whereby virus replication was limited to a single cycle by addition of dextran sulfate (50 μg/mL). The titers were normalized relative to particle numbers as assessed by an RT activity assay and are presented relative to WT (set to 1). Also see Fig. S1 for titer and RT activity values prior to normalization. The columns represent the average of 5–6 independent biological replicates, and the error bars represent standard error of the mean (SEM) (****, *P* < 0.0001; **, *P* < 0.01; *, *P* < 0.05, by one-way ANOVA multiple comparison test with Dunnett’s correction).

The IN R269A/K273A class II substitutions obstruct IN-gRNA binding directly without interfering with IN multimerization and result in the formation of particles with eccentric morphology (15, 16). To gain insight into the molecular basis of how R269 and K273 residues mediate IN-gRNA binding, HIV-1_NL4-3 IN (R269A/K273A)_ class II IN mutant virus was serially passaged in MT-4 T-cells until the emergence of compensatory mutations at the end of passage 3, which resulted in virus growth with wild-type (WT) replication kinetics (Fig. 1A). Deep sequencing of full-length gRNA isolated from virions across the three passages revealed that the IN R269A and K273A substitutions were retained while the nearby D256N and D270N mutations within IN-CTD were sequentially acquired at the end of passage 1 and passage 2, respectively (Fig. 1B). Viruses bearing both mutations dominated cultures by the end of passage 2, and no other nonsynonymous mutations were observed in IN or elsewhere on the gRNA by the end of passage 3.

10.1128/mbio.00431-22.1 FIG S1 Effect of compensatory D256N and D270N substitutions on viral gene expression, Gag processing, particle release, and infectivity for the WT and HIV-1_NL4-3 IN (R269A/K273A)_ class II mutant virus. HEK293T cells were transfected with full-length proviral pNL4-3 expression plasmids carrying mutations coding for the IN D256N, D270N, and D256N/D270N (D2N) substitutions introduced on the WT IN and IN R269A/K273A backbones as described in Fig. 1C. Cell lysates and purified virions were analyzed by immunoblotting for CA and IN. Quantification of unprocessed Gag-Pr55 (A), Gag processing intermediates (B–D), and virion-associated IN levels (E) from 4 independent biological replicate experiments are shown. Error bars show the SEM. (F, G) Cell culture supernatants containing viruses were collected and used in an RT activity assay (F) or titered on TZM-bl indicator cells (G), whereby virus replication was limited to a single cycle by addition of dextran sulfate (50 μg/mL). The RT activity and titer values were normalized relative to WT (set to 1). Download FIG S1, TIF file, 2.6 MB. Copyright © 2022 Shema Mugisha et al. 2022 Shema Mugisha et al. https://creativecommons.org/licenses/by/4.0/ This content is distributed under the terms of the Creative Commons Attribution 4.0 International license.

The IN D256N and D270N mutations were introduced into the replication-competent pNL4-3 HIV-1 molecular clone bearing WT IN or IN R269A/K273A. These substitutions in IN had no observable effect on Gag (Pr55) and Gag-Pol expression or processing in cells, particle release and virion-associated IN levels (Fig. 1C, Fig. S1A-F). Introduction of D256N and D270N mutations either individually or together (D2N) into HIV-1_NL4-3_ bearing WT IN did not affect viral titers (Fig. 1D; Fig. S1G in the supplemental material). Remarkably, while the individual D256N and D270N mutations introduced into the HIV-1_NL4-3 IN (R269A/K273A)_ class II IN mutant virus increased virus titers by approximately 4-fold, the D2N substitutions increased virus titers by 30-fold (Fig. 1D; Fig. S1G). Despite no observable differences in multicycle replication kinetics (Fig. 1A), HIV-1_NL4-3 IN (R269A/K273A/D2N)_ virus appeared ~4-fold less infectious than WT HIV-1 in single cycle replication assays (Fig. 1D; Fig, S1G), likely due to an impact on the catalytic activity of IN as detailed below. Overall, these results demonstrate that the combination of D256N and D270N mutations is sufficient to substantially increase the replication competency of the HIV-1_NL4-3 IN (R269A/K273A)_ class II IN mutant virus.

### IN D256N/D270N substitutions restore IN-gRNA binding and accurate virion maturation for the HIV-1_NL4-3 IN (R269A/K273A)_ virus.

**FIG 2:**
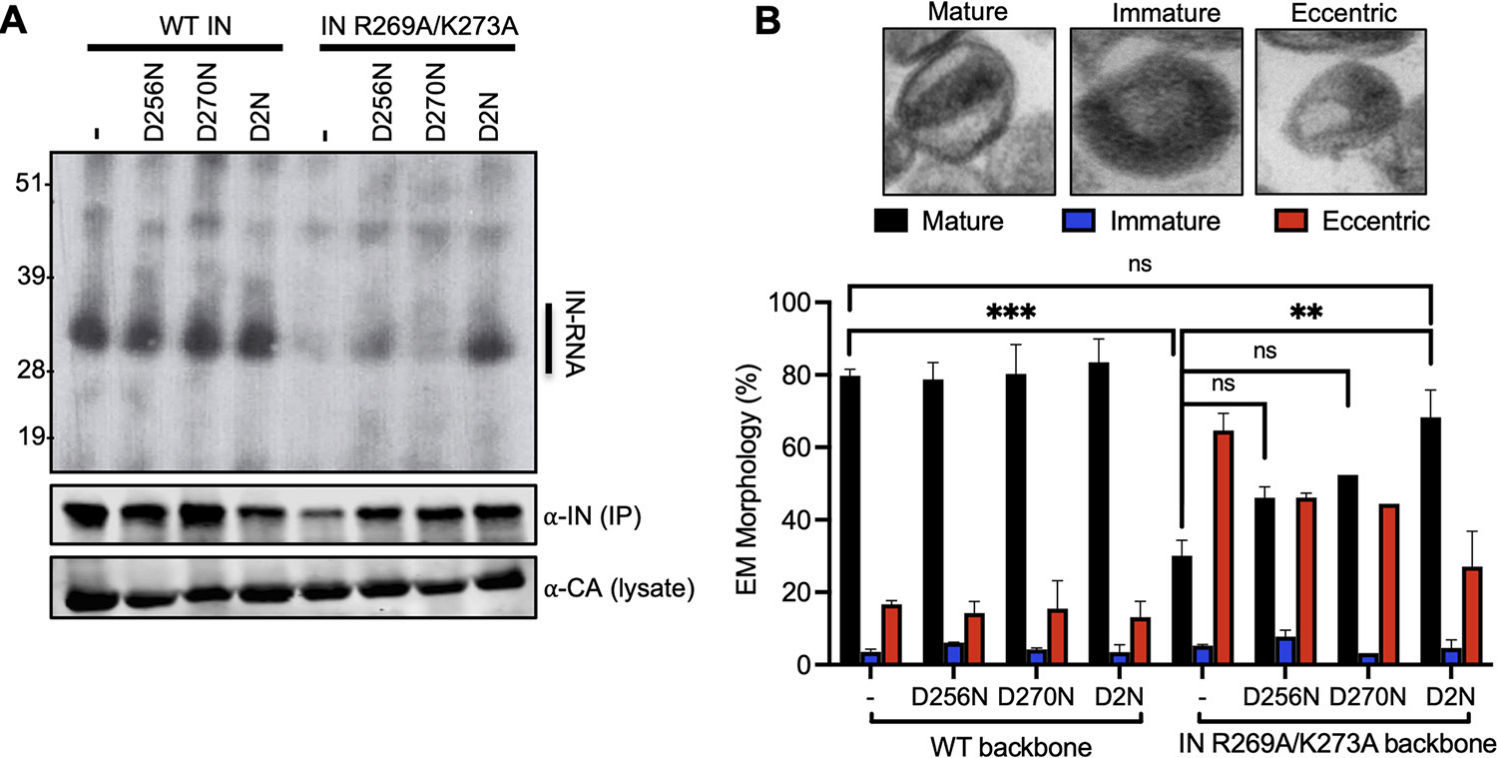
D256N and D270N substitutions restore IN-gRNA binding and accurate virion maturation for the HIV-1_NL4-3 IN (R269A/K273A)_ class II mutant virus. (A) Autoradiogram of IN-RNA adducts immunoprecipitated from virions bearing the indicated substitutions in IN. Immunoblots below show immunoprecipitated (IP) IN or CA protein in lysates prior to immunoprecipitation. Data shown are representative of three independent experiments. See also Fig. S2 for quantitative analysis of autoradiographs. (B) Examination of virion maturation in WT and IN mutant viruses by thin section electron microscopy (TEM). Data show quantification of virion morphologies across 100 particles for each sample and replicate experiment. Data show the average of two independent biological replicates, and error bars represent the SEM (***, *P* < 0.001; **, *P* < 0.01; ns = not significant, by one-way ANOVA multiple comparison test with Dunnett’s correction).

We next assessed whether the IN D256N and IN D270N substitutions rendered the HIV-1_NL4-3 IN (R269A/K273A)_ virus replication competent by restoring IN-gRNA binding and proper virion maturation. To this end, IN-gRNA complexes were immunoprecipitated from UV-cross-linked virions and visualized per CLIP protocol as described previously (38, 74, 75). IN-RNA complexes were readily visible for viruses bearing WT IN, and the D256N, D270N, and D2N substitutions introduced on the WT IN backbone did not impact RNA binding (Fig. 2A and Fig. S2). Expectedly (15, 38), the IN R269A/K273A class II mutant had substantially lower levels of IN-gRNA complexes isolated from virions (Fig. 2A; Fig. S2). The D256N substitution modestly enhanced the ability of IN R269A/K273A to bind RNA, whereas the D270N substitution had no observable impact (Fig. 2A; Fig. S2). In contrast, the D2N substitution substantially enhanced the ability of IN R269A/K273A to bind RNA (Fig. 2A; Fig. S2) and resulted in a significant increase in the accumulation of early and late reverse transcription (RT) products (Fig. S3A, B). The virion morphology of WT and IN mutant viruses was assessed by transmission electron microscopy (TEM). As expected, more than 80% of WT particles had an electron-dense condensate that represents vRNPs inside the CA lattice, whereas the majority of IN R269A/K273A class II mutant virions (~68%) had a clear eccentric morphology (Fig. 2B). Consistent with effects on virus titers and RNA-binding, the introduction of D2N substitutions largely restored the ability of the HIV-1_NL4-3 IN (R269A/K273A)_ virus to form properly matured virions (Fig. 2B). Cumulatively, these data show that D256N and D270N substitutions restore infectivity for the HIV-1_NL4-3 IN (R269A/K273A)_ class II IN mutant virus through reestablishing gRNA binding and thereby enabling proper virion maturation and subsequently reverse transcription in target cells.

10.1128/mbio.00431-22.2 FIG S2 D256N and D270N substitutions restore RNA binding for the HIV-1_NL4-3 IN (R269A/K273A)_ class II mutant virus. IN-RNA adducts were immunoprecipitated from virions bearing the indicated substitutions in IN as in Fig. 2A. Autoradiography images were quantified for IN-RNA adducts and presented relative to WT (set to 1). Data are derived from three independent biological replicates with error bars showing the SEM. (****, *P* < 0.0001; ns = not significant, by one-way ANOVA multiple comparison test with Dunnett’s correction). Download FIG S2, TIF file, 2.7 MB. Copyright © 2022 Shema Mugisha et al. 2022 Shema Mugisha et al. https://creativecommons.org/licenses/by/4.0/ This content is distributed under the terms of the Creative Commons Attribution 4.0 International license.

10.1128/mbio.00431-22.3 FIG S3 Effect of compensatory substitutions on early and late reverse transcription, 2-LTR accumulation. and integration. MT4-LTR-GFP cells were infected with HIV-1_NL4-3_ at an MOI of 0.5 i.u./cell or an equivalent particle number (based on reverse transcriptase [RT] activity) of the class II IN mutant viruses or derivatives bearing compensatory substitutions. Total cell-associated DNA was isolated and subjected to qPCR to quantify early RT products (A), late RT products (B), 2-LTR circles (C), and integration (D) as detailed in Materials and Methods. Data are derived from 3–4 independent replicates, and error bars show the SEM. Download FIG S3, TIF file, 1.4 MB. Copyright © 2022 Shema Mugisha et al. 2022 Shema Mugisha et al. https://creativecommons.org/licenses/by/4.0/ This content is distributed under the terms of the Creative Commons Attribution 4.0 International license.

### A single charge reversal substitution alone enhances IN-gRNA binding and virion infectivity for the HIV-1_NL4-3 IN (R269A/K273A)_ virus.

**FIG 3:**
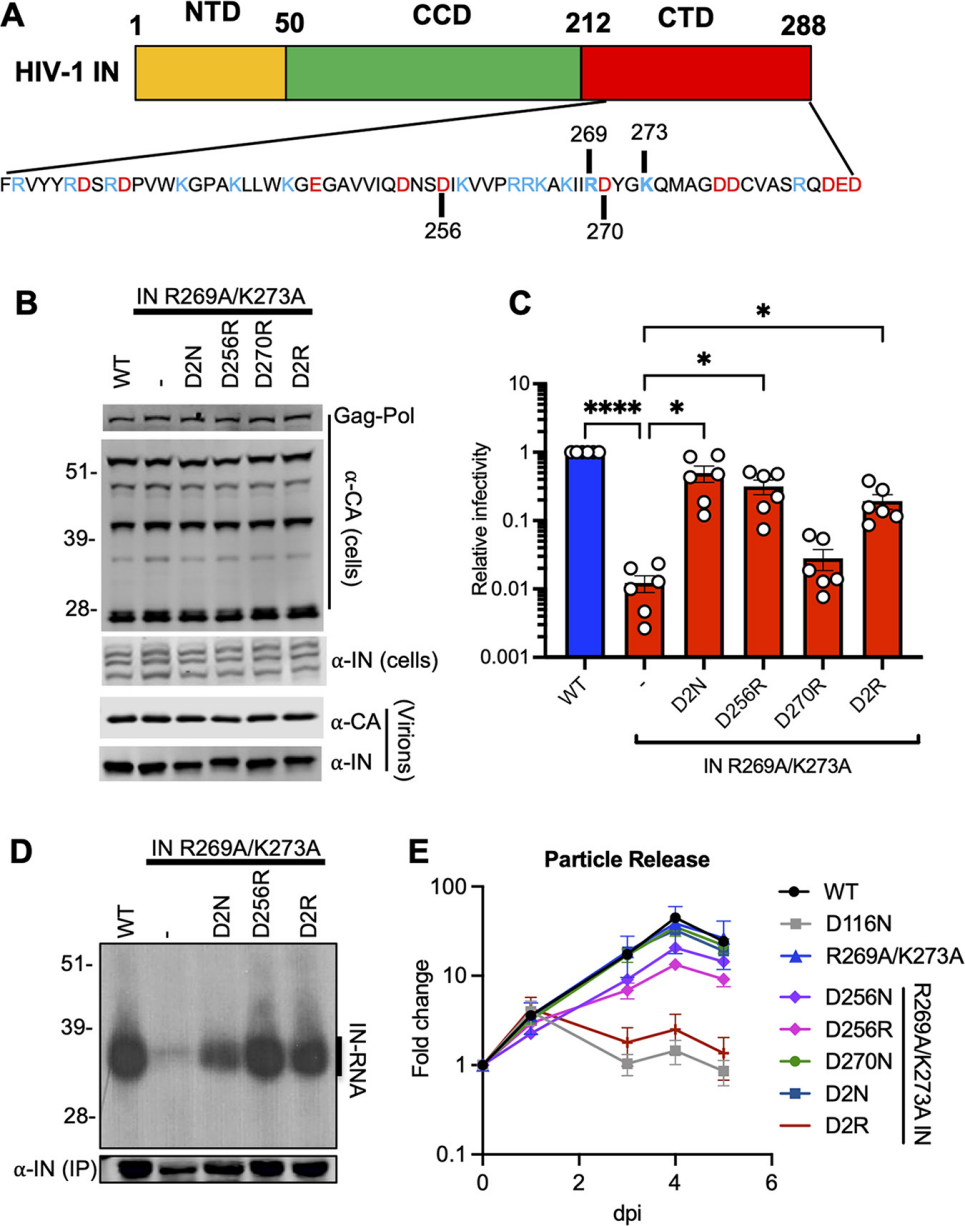
Restoring the local net charge of IN-CTD restores RNA binding and infectivity for the IN R269A/K273A class II mutant. (A) Schematic diagram of IN and sequence of CTD residues with basic and acidic amino acids highlighted in blue and red, respectively. (B, C) HEK293T cells were transfected with full-length proviral WT HIV-1_NL4-3_ expression plasmid or its derivatives carrying *pol* mutations encoding for D2N, D256R, D270R, and D256R/D270R (D2R) IN substitutions on the backbone of HIV-1_NL4-3 IN (R269A/K273A)_. (B) Cell lysates and purified virions harvested 2 days posttransfection were analyzed by immunoblotting for CA and IN. Representative image from five independent experiments is shown. See also Fig. S4A–E for quantitative analysis of immunoblots. (C) Cell culture supernatants containing viruses were titered on TZM-bl indicator cells, whereby virus replication was limited to a single cycle by addition of dextran sulfate (50 μg/mL). The titers were normalized relative to particle numbers as assessed by an RT activity assay and are presented relative to WT (set to 1). Also see Fig. S4F, G for titer and RT activity values prior to normalization. The columns represent the average of six independent experiments, and the error bars represent SEM (****, *P* < 0.0001; *, *P* < 0.05, by one-way ANOVA multiple comparison test with Dunnett’s correction). (D) Autoradiogram of IN-RNA adducts immunoprecipitated from HIV-1_NL4-3_ virus particles bearing WT IN or the indicated IN mutants. Immunoblot below shows immunoprecipitated (IP) IN protein. Results are representative of three independent replicates. See Fig. S4H for quantitative analysis of autoradiographs. (E) MT4 T-cells were infected with HIV-1_NL4-3 IN (D116N)_ viruses that were transcomplemented with the indicated Vpr-IN mutant proteins as described in Materials and Methods. Virion release was assessed by RT activity assays over the course of 5 days postinfection. *y* axis indicates fold increase in RT activity over day 0. Data are from 3 independent replicates; error bars show the SEM.

IN-CTD is decorated with several acidic and basic amino acids, and a long stretch of basic residues spanning 258–273 residues is notable (Fig. 3A). In effect, the D2N substitutions restored the overall local charge of this region, suggesting that electrostatic interactions may be a key parameter in gRNA binding. To test this hypothesis, we investigated whether restoring the overall charge of IN-CTD through other mutations would suppress the class II phenotype observed with HIV-1_NL4-3 IN (R269A/K273A)_. We focused our initial analysis on D256 and D270 residues for the following reasons: (i) these amino acids were amenable to substitutions during virus passaging experiments; and (ii) D256N and D270N substitutions in the context of WT HIV-1 yielded infectious virions, suggesting that these compensatory mutations do not significantly contribute to the catalytic activity of IN, thus allowing us to specifically probe their roles for IN-gRNA interactions and virion maturation. We introduced D256R, D270R, and D256R/D270R (D2R) substitutions into the HIV-1_NL4-3 IN R269A/K273A_ backbone and transfected HEK293T cells with the resulting plasmids. Cell lysates and cell-free virions were then analyzed for Gag and Gag-Pol processing, particle release, and infectivity. Overall, d-to-R substitutions had no major effect on Gag or Gag-Pol expression and processing in cells, or virion release and virion-associated IN levels (Fig. 3B; Fig. S4A–F).

10.1128/mbio.00431-22.4 FIG S4 Effect of compensatory D256R and D270R substitutions on viral gene expression, Gag processing, particle release, infectivity, and IN-RNA binding for the WT and HIV-1_NL4-3 IN (R269A/K273A)_ class II mutant viruses. HEK293T cells were transfected with full-length proviral pNL4-3 expression plasmids carrying mutations coding for the IN D2N, D256R, D270R, and D256R/D270R (D2R) substitutions introduced on the IN R269A/K273A backbone as described in Fig. 3B and C. Cell lysates and purified virions were analyzed by immunoblotting for CA and IN. Quantification of unprocessed Gag-Pr55 (A), Gag processing intermediates (B–D), and virion-associated IN levels (E) from 4 independent biological replicate experiments are shown. Error bars show the SEM. (F, G) Cell culture supernatants containing viruses were collected and used in an RT activity assay (F) or titered on TZM-bl indicator cells (G), whereby virus replication was limited to a single cycle by addition of dextran sulfate (50 μg/mL). The RT activity and titer values were normalized relative to WT (set to 1). (H) IN-RNA adducts were immunoprecipitated from virions bearing the indicated substitutions in IN as in Fig. 3D. Autoradiography images were quantified for IN-RNA adducts and presented relative to WT (set to 1). Data are derived from three independent biological replicates with error bars showing the SEM. (***, *P* < 0.001; **, *P* < 0.01, by one-way ANOVA multiple comparison test with Dunnett’s correction). Download FIG S4, TIF file, 1.8 MB. Copyright © 2022 Shema Mugisha et al. 2022 Shema Mugisha et al. https://creativecommons.org/licenses/by/4.0/ This content is distributed under the terms of the Creative Commons Attribution 4.0 International license.

Remarkably, the D256R substitution alone increased the titers of HIV-1_NL4-3 IN (R269A/K273A)_ by 26-fold to a level comparable with the D2N substitution (Fig. 3C; Fig. S4G). In contrast, the D270R substitution did not increase virus titers and the D2R substitution increased the infectivity of HIV-1_NL4-3 IN (R269A/K273A)_ relatively modestly (~13-fold) compared to D256R and D2N substitutions (Fig. 3C, Fig. S4G). Both the D256R and the D2R substitutions restored IN-gRNA binding to WT levels (Fig. 3D; Fig. S4H) and increased the accumulation of early and late RT products (Fig. S3A, B). These findings demonstrate that the increase in viral titers with the D256R and D2R substitutions correlates well with enhancement of IN-gRNA binding and subsequently reverse transcription.

To determine whether the D256N/R and D270N/R substitutions affected the catalytic activity of IN, we conducted a transcomplementation assay that relies on incorporation of class II IN mutants into a class I IN mutant virus (HIV-1_NL4-3 IN (D116N)_) bearing a catalytically inactive IN through fusion to the viral Vpr protein (76). In this assay, the RNA-binding defect of a class II IN mutant is complemented in *trans* by the catalytically inactive IN, whereas the class II IN mutant can complement the catalytic activity defect of the class I mutant. This allows the specific assessment of class II and compensatory substitutions on the catalytic activity of IN, bypassing the need for successful completion of steps prior to integration. We found that the D256R substitution modestly decreased and the D2R substitutions completely abolished the ability of the R269A/K273A mutant to transcomplement the catalytically inactive IN D116N (Fig. 3E). None of the other IN substitutions appeared to have an impact. Interestingly, within the context of full-length viruses, we found that despite restoring reverse transcription to WT levels, the D2N substitution led to higher levels of 2-LTR accumulation and a 5-fold defect in integration (Fig. S3C, D). Similarly, D256R and D2R substitutions resulted in a modestly higher level of 2-LTR accumulation and a significantly lower level of integration (Fig. S3A–D). Taken together, these findings indicate that despite a decrease in the catalytic activity of IN, restoring the overall charge of IN-CTD through D2N and D256R substitutions specifically reestablishes RNA binding, overcomes the reverse transcription defect, and increases virion infectivity for the IN R269A/K273A class II mutant.

### Charge reversal substitutions suppress the replication defect of a separate class II IN mutant virus through restoring gRNA binding.

**FIG 4:**
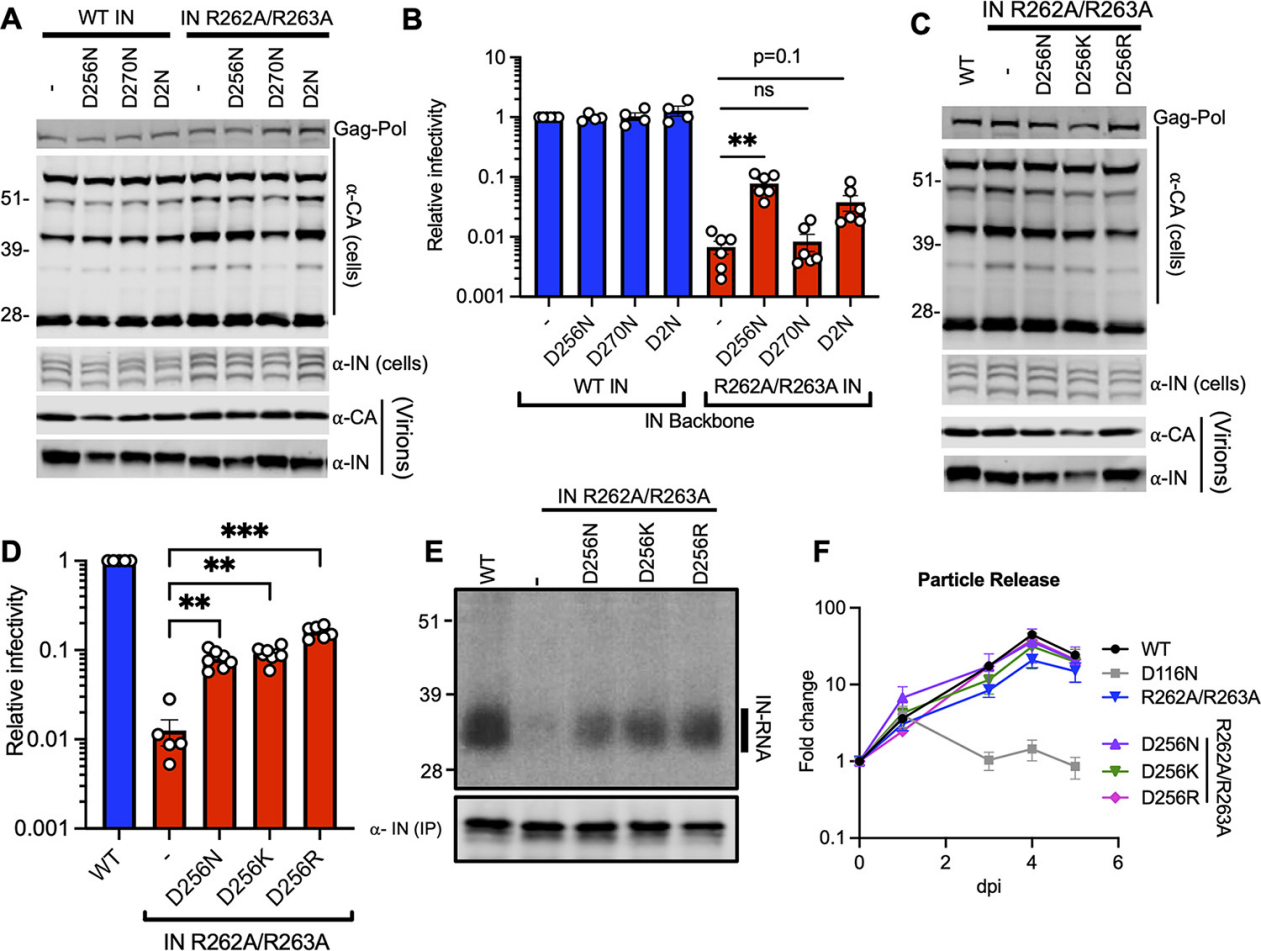
D256R and D256K substitutions restore IN-RNA binding and infectivity for the HIV-1_NL4-3 IN (R262A/R263A)_ class II mutant virus. (A–E) HEK293T cells were transfected with proviral HIV-1_NL4-3_ expression plasmids carrying *pol* mutations for the indicated IN substitutions on WT IN or IN R262A/R263A backbones. (A, C) Cell lysates and purified virions collected 2 days posttransfection were analyzed by immunoblotting for CA and IN. See also Fig. S5 for quantitative analyses of immunoblots. (B, D) Cell culture supernatants containing viruses were titered on TZM-bl indicator cells, whereby virus replication was limited to a single cycle by addition of dextran sulfate (50 μg/mL). The titers were normalized relative to particle numbers as assessed by an RT activity assay and are presented relative to WT (set to 1). See also Fig. S5F, L, M for titer and RT activity values prior to normalization. The columns represent the average of four-to-six independent experiments, and the error bars represent SEM (**, *P* < 0.01; *, *P* < 0.05; ns = not significant by one-way ANOVA multiple comparison test with Dunnett’s correction). (E) Autoradiogram of IN-RNA adducts immunoprecipitated from WT or IN mutant HIV-1_NL4-3_ virions. The amount of immunoprecipitated IN was assessed by the immunoblot shown below. Immunoblots and CLIP autoradiographs are representative of three independent replicates. See Fig. S5N for quantitative analysis of autoradiographs. (F) MT4 T-cells were infected with HIV-1_NL4-3 IN (D116N)_ viruses that were transcomplemented with the indicated Vpr-IN mutant proteins as described in Materials and Methods. Virion release was assessed by RT activity assays over the course of 5 days postinfection. *y* axis indicates fold increase in RT activity over day 0. Data are from 3 independent replicates; error bars show the SEM.

Mutation of other basic residues within the IN-CTD, such as R262A/R263A, also directly inhibits IN-gRNA binding without compromising oligomerization of IN (15, 38). If IN-gRNA binding is mediated by electrostatic interactions, we reasoned that substitutions of D256 and D270 residues in a way that restores the local positive charge should restore IN-gRNA binding and infectivity in the background of HIV-1_NL4-3 IN (R262A/R263A)_. D256N, D256K, D256R, D270N, and D2N substitutions introduced into HIV-1_NL4-3 IN (R262A/R263A)_ did not have a major impact on Gag and Gag-Pol expression, though a modest increase in distinct Gag processing intermediates was observed (Fig. 4A, C; Fig. S5A–D, G–J). Notwithstanding, neither virion release nor virion-associated IN levels were impacted (Fig. 4A, C; Fig. S5E, K, L). IN D256N/K/R substitutions increased virion infectivity by 10–13-fold, D2N substitutions 5-fold, whereas the D270N substitution had no impact (Fig. 4B, D; Fig. S5F, M). In line with the titer data, D256N/K/R substitutions all significantly increased IN-gRNA binding and accumulation of early and late RT products (Fig. 4E; Fig. S3A, B; Fig. S5N). D256N/K/R IN successfully transcomplemented a class I IN mutant (D116N) but displayed catalytic activity defects within the context of full-length viruses (Fig. S3C, D), suggesting that they specifically restored the second noncatalytic function of IN, namely, gRNA binding (Fig. 4F).

10.1128/mbio.00431-22.5 FIG S5 Effect of compensatory substitutions on viral gene expression, Gag processing, particle release, infectivity, and IN-RNA binding for the WT and HIV-1_NL4-3 IN (R262A/R263A)_ class II mutant viruses. HEK293T cells were transfected with full-length proviral pNL4-3 expression plasmids carrying mutations coding for the IN D256N, D270N, D2N, D256K, and D256R substitutions introduced on the WT IN or IN R262/R263A backbones as described in Fig. 4. Cell lysates and purified virions were analyzed by immunoblotting for CA and IN. Quantification of unprocessed Gag-Pr55 (A, G), Gag processing intermediates (B, C, D, H, I, J), and virion-associated IN levels (E, K) from 4 independent biological replicates are shown. (F, L, M) Cell culture supernatants containing viruses were collected and titered on TZM-bl indicator cells (F, M) or used in an RT activity assay (L) to assess particle release. The RT activity and titer values were normalized relative to WT (set to 1). (N) IN-RNA adducts were immunoprecipitated from virions bearing the indicated substitutions in IN as in Fig. 4E. Autoradiography images were quantified and presented relative to WT (set to 1). Data are derived from three independent biological replicates, with error bars showing the SEM. (****, *P* < 0.0001; **, *P* < 0.01; *, *P* < 0.05; ns = not significant, by one-way ANOVA multiple comparison test with Dunnett’s correction). Download FIG S5, JPG file, 1.0 MB. Copyright © 2022 Shema Mugisha et al. 2022 Shema Mugisha et al. https://creativecommons.org/licenses/by/4.0/ This content is distributed under the terms of the Creative Commons Attribution 4.0 International license.

### Charge reversal substitutions at acidic residues other than D256 can suppress the class II phenotype.

**FIG 5:**
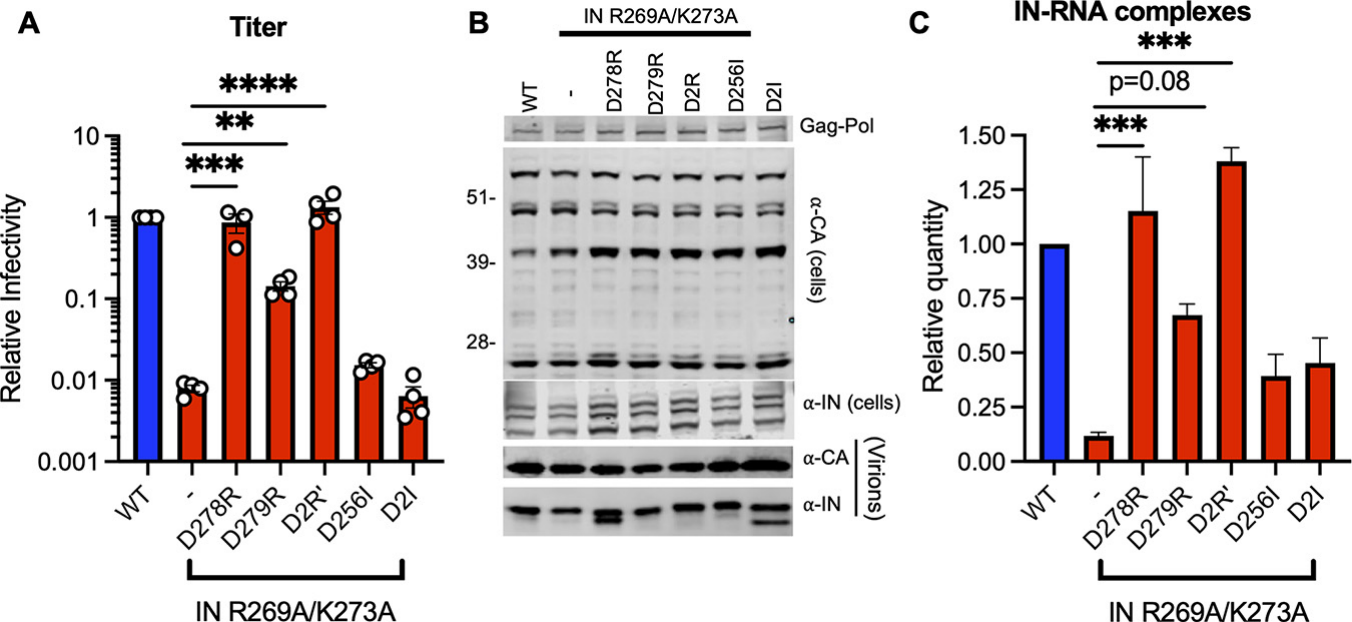
D279R substitution increases and D278R and D2R’ substitutions restore IN-RNA binding and infectivity for the HIV-1_NL4-3 IN (R269A/K273A)_ virus. HEK293T cells were transfected with full-length pNL4-3 expression plasmids carrying mutations coding for the IN D278R, D279R, D278R/D279R (D2R’), D256I, and D256I/D270I (D2I) substitutions introduced on the IN R269A/K273A backbone. (A) Cell culture supernatants containing viruses were titered on TZM-bl indicator cells, whereby virus replication was limited to a single cycle by addition of dextran sulfate (50 μg/mL). The titers were normalized relative to particle numbers by an RT activity assay and are presented relative to WT (set to 1). The columns represent the average of 4 independent biological replicates, and the error bars represent standard error of the mean (SEM) (*, *P* < 0.05, by one-way ANOVA multiple comparison test with Dunnett’s correction). (B) Cell lysates and purified virions collected 2 days posttransfection were analyzed by immunoblotting for CA and IN. The image is representative of four independent experiments. (C) IN-RNA adducts immunoprecipitated from WT or IN mutant HIV-1_NL4-3_ virions per CLIP protocol were analyzed by autoradiography. Graph shows the quantification of IN-RNA adducts from three independent biological replicates; error bars show the SEM.

Given the above findings, we next determined whether charge reversal substitutions at other nearby acidic residues, such as D278 and D279, could restore IN-gRNA binding and infectivity for the HIV-1_NL4-3 IN (R269A/K273A)_ virus. Remarkably, while the D279R substitution increased infectivity by 18-fold, the D278R substitution and the D278R/D279R (D2R’) substitutions completely restored infectivity to WT levels (Fig. 5A). We also tested whether substitutions of the original D256 and D270 residues into Ile would restore virus titers at levels similar to the d-to-N substitutions. However, we found that neither D256I alone nor the D256I/D270I (D2I) substitutions increased viral titers (Fig. 5A). Of note, these substitutions did not impact Gag and Gag-Pol expression, processing, virion release, or virion-associated IN levels, though we noted the presence of an aberrantly processed IN with the D278R and D2I mutants (Fig. 5B). In line with the titer data, D279R substitution increased and the D278R and D2R’ substitutions restored IN-RNA binding for the HIV-1_NL4-3 IN (R269A/K273A)_ virus (Fig. 5C). Subsequently, D278R and D2R’ substitutions substantially increased the accumulation of reverse transcription products (Fig. S3A, B) without impairment of IN catalytic activity as evident in lack of 2-LTR accumulation (Fig. S3C) and near WT levels of integration (Fig. S3D). These and above results demonstrate that restoring the local positive charge of IN-CTD can efficiently restore/enhance RNA binding, reverse transcription, and virion infectivity to two distinct class II IN mutants. Cumulatively, these findings strongly suggest the importance of electrostatic interactions in mediating IN-gRNA binding.

### Compensatory substitutions directly and specifically restore the ability of IN to bind vRNA.

**FIG 6:**
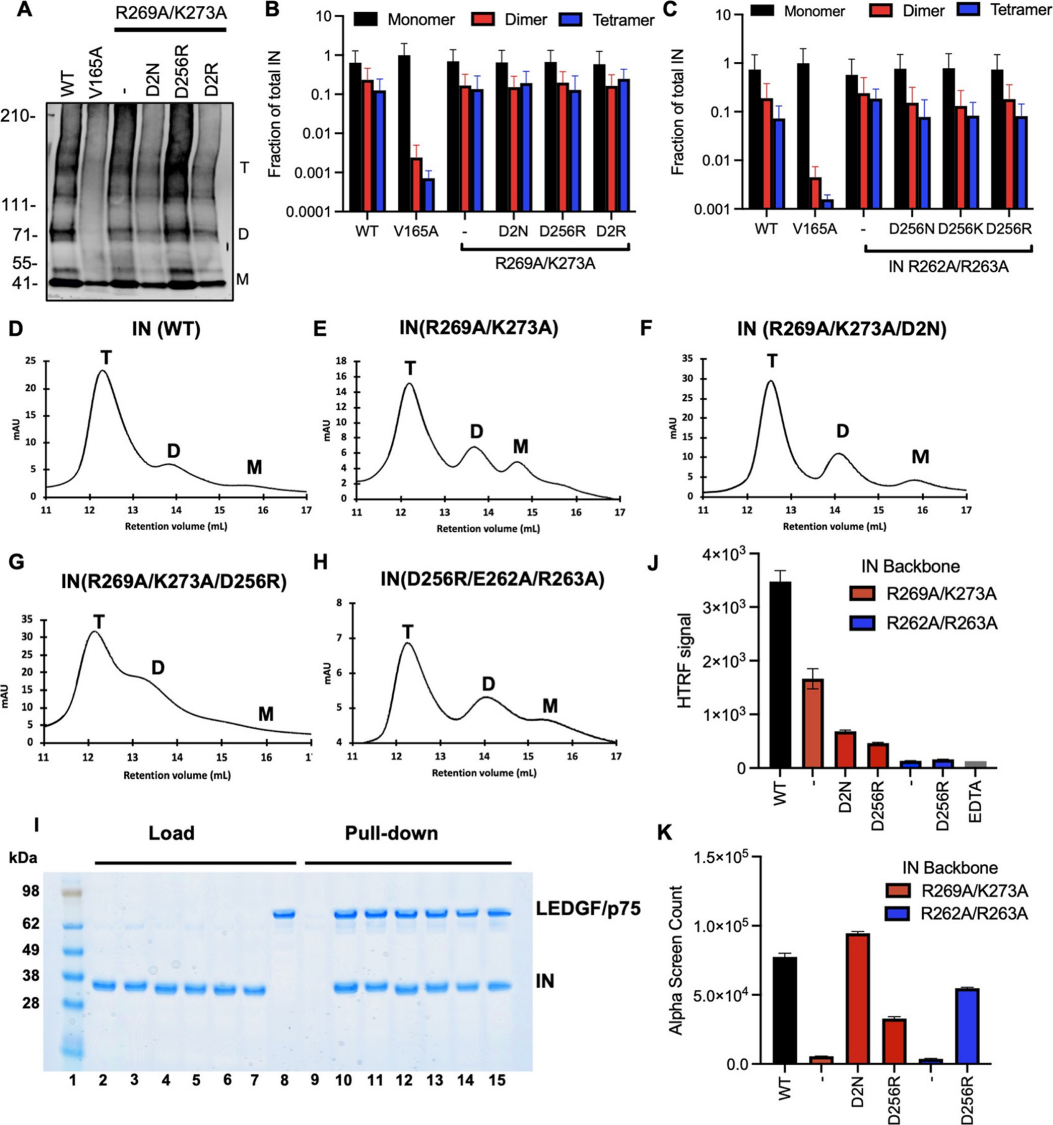
Assessing multimerization properties of IN in mutant viruses. (A) Purified WT or IN mutant HIV-1_NL4-3_ virions were treated with 1 mM EGS, and virus lysates analyzed by immunoblotting using antibodies against IN following separation on 3–6% Tris-acetate gels as detailed in Materials and Methods. The position of monomers (M), dimers (D), and tetramers (T) are indicated by arrows in a representative Western blot. (B, C) Quantification of IN multimerization in virions from experiments conducted as in A. Error bars show the SEM from three independent experiments. (D–H) Representative SEC traces for indicated recombinant IN proteins at 20 μM. The *x* axis indicates elution volume (mL) and *y* axis indicates the intensity of absorbance (mAU). Tetramers (T), dimers (D), and monomers (M) are indicated. Representative chromatograms from two independent analyses are shown. (I) Affinity-pulldown assays showing binding of WT and mutant INs to LEDGF/p75. Lane 1: molecular weight marker; Lanes 2–8: loads of 6xHis-tagged WT IN, IN(R269A/K273A), IN(R269A/K273A/D256N/D270N), IN(R269A/K273A/D256R), IN(D256R/R262A/R263A), IN(D256R/R262A/R263A), and FLAG-tagged LEDGF/p75; Lanes 9–15: affinity pull-down using Ni beads of LEDGF/p75 with buffer only (control), 6xHis-tagged WT IN, IN(R269A/K273A), IN(R269A/K273A/D256N/D270N), IN(R269A/K273A/D256R), IN(D256R/R262A/R263A), and IN(D256R/R262A/R263A). (J) Catalytic activities of mutant IN molecules in the presence of LEDGF/p75 monitored by HTRF based assay. The bars represent the ratio of emission signal at 665 nm over that of 615 nm, which was then multiplied by a factor of 10,000. The error bars indicate the standard error of the mean from triplicate experiments. (K) Summary of mutant INs bridging TAR RNA compared to WT IN. Alpha screen counts at 320 nM for each protein is shown. The graphs show average values of three independent experiments, and the error bars indicate standard deviation.

As IN oligomerization is a prerequisite for RNA binding (15), we next examined how the compensatory substitutions affected IN oligomerization. For in-virion analysis, purified HIV-1_NL4-3_ virions (WT and bearing the relevant IN substitutions) were treated with ethylene glycol bis (succinimidyl succinate) (EGS) to covalently cross-link IN *in situ*, and virus lysates were analyzed by immunoblotting. As in WT virions, IN species that migrated at molecular weights consistent with those of monomers, dimers, trimers, and tetramers were readily distinguished in HIV-1_NL4-3 IN (R269A/K273A)_ and HIV-1_NL4-3 IN (R262A/R263A)_ viruses with additional compensatory mutations (D256N/D270N, D256R, D256K, and D256R/D270R) but not with the canonical class II IN mutant V165A that is unable to form functional oligomers (Fig. 6A to C; reference 15).

Complementary *in vitro* experiments assessed oligomeric states of purified recombinant IN proteins by size exclusion chromatography (SEC). Results in Fig. 6D to H show that while tetramer/dimer/monomer ratios varied for different mutant proteins, the tetrameric form was a predominant species for all analyzed proteins under our experimental conditions. We also examined the ability of IN mutant proteins to bind LEDGF/p75, which preferentially interacts with and stabilizes IN tetramers (77). The results in Fig. 6I show that all tested proteins effectively interacted with LEDGF/p75. Collectively, the results in Fig. 6D–I suggest that D2N and D256R substitutions in the background of IN R269A/K273A or the IN R262A/R263A class II mutants did not grossly alter IN tetramerization.

Next, we examined the ability of the mutant proteins to perform two critical functions *in vitro*: (i) catalyze integration (Fig. 6J), and (ii) bind and bridge synthetic RNA (Fig. 6K). Consistent with our previous findings (38), R269A/K273A substitutions only modestly (up to 2-fold) affected the catalytic function of the recombinant protein (Fig. 6J), whereas these substitutions abolished binding of the mutant protein to the synthetic TAR RNA (Fig. 6K). Strikingly, additional D2N substitutions engineered in the background of R269A/K273 IN exhibited opposite effects on integration activity versus RNA interactions. In line with infection-based experiments (Fig. S3), these additional substitutions further reduced the catalytic activity of the parental R269A/K273 mutant (Fig. 6J). IN R269A/K273A/D2N was ~5-fold less active than its WT counterpart (Fig. 6J) but exhibited WT levels of RNA binding (Fig. 6K). These biochemical results suggest that the D2N substitutions specifically restore the ability of the parental R269A/K273A IN to bind RNA. Similarly, the D256R substitution specifically enhanced the abilities of IN R269A/K273A and IN R262A/R263A to bridge between RNA molecules (Fig. 6K) without substantially affecting catalytic activities of these proteins. Taken together, the biochemical results in Fig. 6 demonstrate that the compensatory mutations directly and specifically restore the ability of IN to bind RNA.

### Characterization of IN mutations present in latently infected cells.

**FIG 7:**
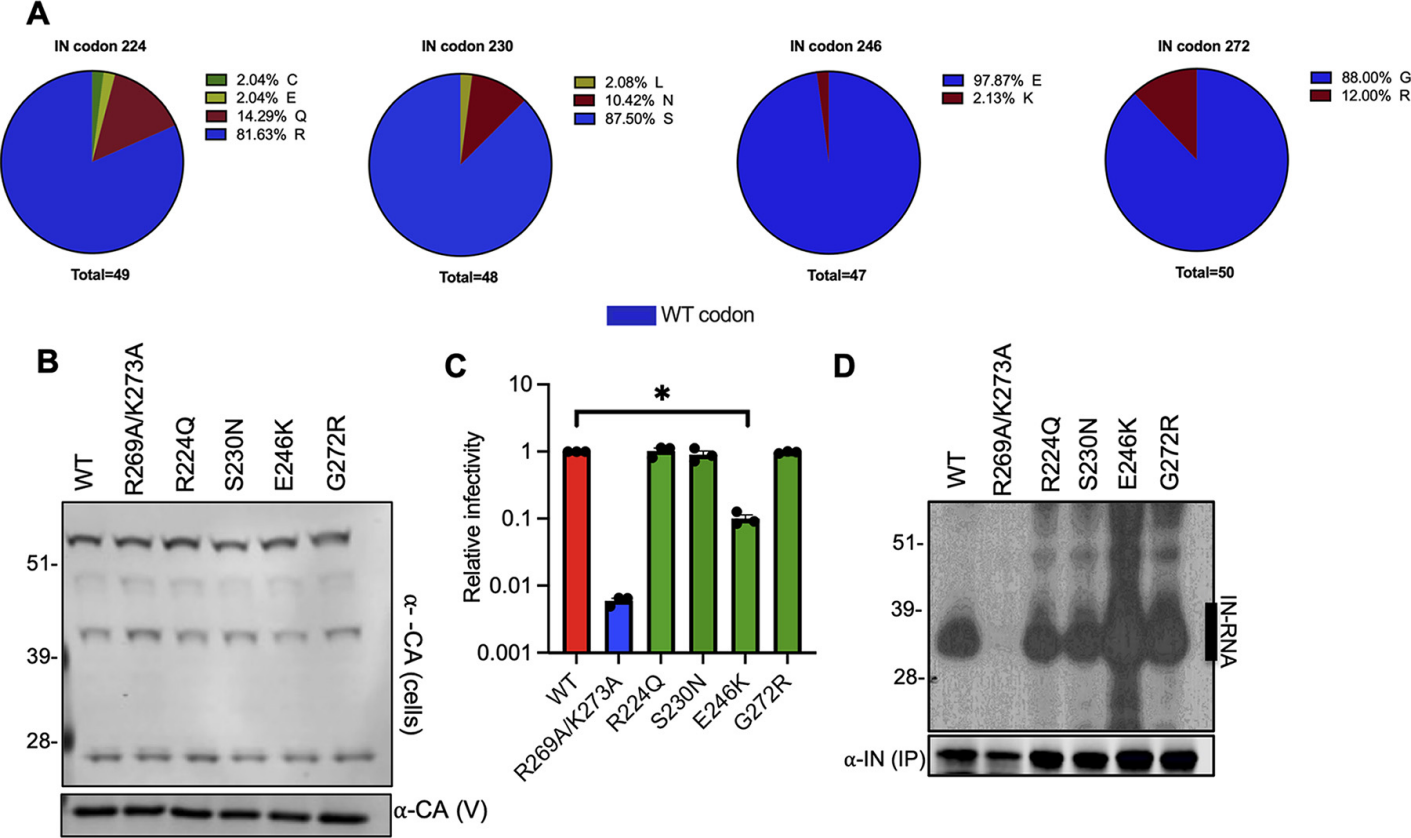
Characterization of IN mutations present in latently infected CD4^+^ T cells. (A) Frequency of IN substitutions at amino acids 224, 230, 246, and 272 derived from HIV-1 sequences from latently infected CD4^+^ T-cells is shown. (B) HEK293T cells were transfected with proviral HIV-1_NL4-3_ expression plasmids carrying the R224Q, S230N, E246K, and G272R IN mutations. Cell lysates and virions were purified 2 days posttransfection and analyzed by immunoblotting for CA and IN. The image is representative of two independent experiments. (C) WT or IN mutant HIV-1L4-3 viruses in cell culture supernatants were titered on TZM-bl indicator cells. The titers are presented relative to WT (set to 1). The columns represent the average of three independent experiments, and the error bars represent SEM (*, *P* < 0.05, by one-way ANOVA multiple comparison test with Dunnett’s correction). (D) Autoradiogram of IN-RNA adducts immunoprecipitated from virions bearing the indicated substitutions in IN. Immunoblots below show the amount of immunoprecipitated IN.

Persistence of HIV-1 in memory CD4^+^ T-cells as latent proviruses constitutes a major barrier to HIV-1 cure. Although the majority of HIV-1 proviruses in these cells are defective (78), recent evidence suggests that defective proviruses can be transcribed into RNAs that are spliced and translated, and can be recognized by HIV-1-specific cytotoxic T lymphocytes (79). We decided to characterize IN mutations isolated from latently infected cells, given the possibility that class II IN mutations existing in latently infected cells can result in the formation of defective particles that may subsequently modulate immune responses. Though relatively uncommon, we found the presence of R224Q, S230N, E246K, and G272R substitutions in IN-CTD (Fig. 7A). Of note, only the R224Q substitution resulted in loss of a positive charge, whereas the E246K and G273R substitutions resulted in gain of positive charges. These mutations were introduced into the NL4-3 proviral backbone with minimal effects on Gag expression and particle release (Fig. 7B). Although the E246K virus was significantly less infectious (Fig. 7C), we did not find any evidence for loss of IN-gRNA binding (Fig. 7D), suggesting that this mutant likely displays a class I phenotype. Thus, we conclude that the class II mutant viruses are rarely present in the latently infected cells and therefore unlikely to contribute to chronic immune activation.

## DISCUSSION

Class II IN mutations impair virion particle maturation by blocking IN-gRNA binding in virions and those within the CTD, including R269A/K273A and R262A/R263A substitutions, impede IN-gRNA binding without affecting oligomerization of IN (15, 38). During serial passaging of the IN R269A/K273A class II mutant virus, the D256N and D270N substitutions emerged sequentially. We also wondered whether mutations outside of IN, such as CA and NC, could also arise, given that the IN R269A/K273A mutant is still catalytically active and compensatory mutations in CA and NC could potentially provide alternative mechanisms for the proper packaging of the gRNA within virions. However, we did not observe the emergence of such substitutions showcasing the distinct role of IN-gRNA binding in proper virion maturation. Furthermore, the emergence of compensatory substitutions only in CTD, but not NTD or CCD of IN, highlights the importance of the IN-CTD in mediating direct RNA binding.

It is worth noting that D256N and D270N substitutions each arose through a single mutation (D256N: GAC→AAC, D270N: GAU→AAU), and thus possibly provided an easier pathway for suppression than reverting back to R269 and K273, each of which would require two mutations. Likewise, d-to-R and d-to-K substitutions require two and three mutations, respectively, and possibly through an unfavorable intermediate amino acid substitution, creating a higher barrier to arise in culture. While the D278R substitution was the most effective in restoring gRNA binding and without any impact on the catalytic activity of IN, the rise of this mutation was likely additionally limited by the necessity to maintain a functional Vif given the overlap of IN-CTD and Vif ORFs. Though other mutations in IN could in principle restore the ability of IN to bind RNA, rise of such mutations was mostly likely constrained in part by the necessity to maintain a catalytically active IN.

The IN-CTD is decorated by numerous acidic and basic residues (Fig. 3A). While the IN R269A/K273A substitutions resulted in the loss of 2 positive charges, the compensatory D2N substitutions restored the overall local charge within the CTD, suggesting that electrostatic interactions contribute to IN-CTD binding to the gRNA. Consistent with this hypothesis, the D256R, D278R, and D279R substitutions that resulted in the gain of two positive charges locally were all sufficient to substantially increase or restore IN-gRNA binding, and subsequently reverse transcription and infectivity for the IN R269A/K273A mutant. Furthermore, these observations extended to another class II mutant, IN R262A/R263A, whereby the D256N/K/R substitutions specifically enhanced RNA binding and subsequently increased the accumulation of RT products and infectivity. In contrast, the D2N substitutions did not enhance RNA-binding or infectivity further for the IN R262A/R263A mutant, demonstrating a degree of context/localization dependency. In other words, it is possible that the proximity of the D270N to R269A/K273A residues may explain why it was more effective in restoring RNA binding particularly for this class II mutant but not for IN R262A/R263A. Cumulatively, our findings revealed a key electrostatic component of IN-gRNA interactions.

Inspection of the available X-ray structure (63) indicates that all IN residues implicated in RNA binding by the present study are positioned either in the highly flexible C-terminal tail (amino acids [aa] 261–275) or the 252–257 loop (Fig. S6H). R269A and K273A substitutions expectedly resulted in a substantial loss of a basic patch in IN (Fig. S6A, B). Both the D256R and D256N substitutions resulted in a more positively charged surface distal from the R269/K273A residues (Fig. S6C, D), suggesting that compensatory mutations likely created an additional interaction interface for gRNA binding. A similar outcome was observed when class II R262A/R263A and compensatory D256R changes were introduced in the CTD of IN (Fig. S6E–G). While the D256R change is substantially distanced from R262/R263 and R269/K273 residues, we note the following. R262, R263, R269, and K273 are positioned within the same highly flexible C-terminal tail (aa 261–275), whereas D256 belongs to another, shorter (aa 252–257) loop (Fig. S6H). The highly pliable nature of the tail and the loop could be crucial for IN to optimally engage with cognate RNA as well as allow for emergence of compensatory mutations at alternative sites positioned in these IN segments.

10.1128/mbio.00431-22.6 FIG S6 Electrostatic potential maps of HIV-1 IN bearing class II and compensatory mutations. (A–G) Electrostatic potential maps of the indicated HIV-1 IN mutants derived from the crystal structure of Gupta et al. (63) are depicted. Results are displayed as an electrostatic potential molecular surface. The low, mid, and high range values are −5, 0, and 5, respectively. (H) A cartoon view of the CTD structure (63) is shown with the C-terminal tail (aa 261–275) and the loop (aa 252–257) colored in cyan and orange, respectively. Side chains of indicated residues are shown. Download FIG S6, TIF file, 1.8 MB. Copyright © 2022 Shema Mugisha et al. 2022 Shema Mugisha et al. https://creativecommons.org/licenses/by/4.0/ This content is distributed under the terms of the Creative Commons Attribution 4.0 International license.

Arg and Lys residues are commonly employed by numerous RNA-binding proteins, and Arg residues are generally more heavily involved interactions with all bases (71, 80–84). Though the electrostatic interactions between the Arg/Lys residues of IN-CTD and gRNA imply a level of nonspecificity, IN binds to distinct locations on the gRNA in virions and displays high binding affinity to structured elements, such as TAR (38). Thus, it is likely that IN-gRNA interactions are mediated by both the nonspecific interactions of the basic residues with the RNA phosphate backbone and specific interactions with the cognate RNA, as has also been suggested by a recent modeling study (73). Importantly, we note that while the present study elucidates crucial roles of the IN-CTD basic residues, other yet-to-be-identified IN amino acids are likely to also contribute to high affinity binding of select gRNA segments. For example, recognition of the TAR loop by Tat and the super elongation complex is based on a complex set of interactions: the super elongation complex primarily reads out the structure as opposed to the sequence, whereas the Tat arginine rich motif (ARM) interacts with the TAR bulge and the major groove through electrostatic interactions with the RNA backbone and H-bonding with specific bases (85, 86). Structural studies of IN in complex with cognate RNA oligonucleotides are crucial to tease apart the specificity determinants for IN-gRNA interactions.

RNA binding proteins commonly encode modular RNA-binding domains (i.e., RRMs and KH domains), which form specific contacts with short degenerate sequences (87–89). Utilization of multiple RRMs/KH domains is thought to create a much larger binding interface, which in turn allows for specific, high-affinity binding to target RNAs (87, 88, 90). Functionally important IN tetramerization (15) may similarly enhance binding affinity and specificity to cognate gRNA elements. In this respect, it is worth noting that CTDs of the two inner protomers in the tetrameric intasome structure contact extensively with DNA, whereas the outer CTDs are positioned in close proximity to the inner CTDs and only partially contribute DNA binding (91). It remains to be seen whether the inner CTDs could also preferentially engage gRNA, or CTD-nucleic acid interactions within IN-gDNA and IN-gRNA complexes differ substantially.

Overall, our studies reveal that pliable electrostatic interactions play an important role in mediating IN-CTD-gRNA interactions and demonstrate that CTD is a key determinant of gRNA binding. IN-gRNA binding is essential for HIV-1 virion morphological maturation and infectivity, thus an excellent target for novel antiretroviral compounds. ALLINI-mediated inhibition of the noncatalytic function of IN can complement existing INSTI-based therapies and increase the barrier to drug resistance substantially. Of note, a highly potent and safe pyrrolopyridine-based ALLINI, STP0404, has recently advanced to human trials (66). Further structural characterization of IN-gRNA and IN-ALLINI complexes will be crucial to determine the precise rules that govern IN-gRNA interactions and guide the development of novel therapeutics that target this noncatalytic function of IN.

## MATERIALS AND METHODS

### Plasmids and compounds.

IN mutations were introduced into the HIV-1_NL4-3_ full-length proviral plasmid (pNL4-3) by overlap extension PCR. Forward and reverse primers containing IN mutations were used in PCRs with forward (with EcoRI restriction endonuclease site) and reverse (with AgeI restriction endonuclease site) outer primers. The resulting fragments containing the desired mutations were mixed at a 1:1 ratio and overlapped subsequently using the outer primer pairs. The overlap fragments were digested with AgeI and EcoRI before cloning into pNL4-3 plasmids. The pLR2P-VprIN plasmid expressing a Vpr-IN fusion protein has been previously described (76). IN mutations were introduced in the pLR2P-VprIN plasmid using the QuikChange site-directed mutagenesis kit (Agilent Technologies). The presence of the desired mutations and the absence of unwanted secondary changes was assessed by Sanger sequencing.

ALLINIs, BI-D and BI-B2, were synthesized by Aris Pharmaceuticals using previously published protocols (92, 93). Compounds were dissolved in dimethyl sulfoxide (DMSO) at a final concentration of 10 mM and stored at −80 °C.

### Cell lines and viruses.

HeLa-derived TZM-bL cells that express CD4/CXCR4/CCR5 receptor/coreceptors and bear an HIV-1 LTR driven β-galactosidase reporter were obtained from the NIH AIDS Reagent Program. HEK293T cells (ATCC CRL-11268) and TZM-bL cells were cultured in Dulbecco’s modified Eagle’s medium supplemented with 10% fetal bovine serum. Human CD4^+^ T-cell line, MT4 (NIH AIDS Reagents), were cultured in RPMI 1640 medium supplemented with 10% fetal bovine serum. A derivative of MT4-LTR-GFP (MT4-GFP) indicator cells bearing an HIV-1 LTR driven GFP reporter have been described before (94) and similarly maintained in RPMI 1640 medium supplemented with 10% fetal bovine serum. All cell lines were obtained from the American Type Culture Collection and NIH AIDS Reagents, where short tandem repeat (STR) profiling was performed. MT-4 T-cells were further authenticated by STR profiling at Washington University School of Medicine Genome Engineering and iPSC center. The cell lines are regularly inspected for mycoplasma contamination using the MycoAlert mycoplasma detection kit (Lonza) and checked for being free of any other contaminations.

For generation of HIV-1 stocks, HEK293T cells grown in 10-cm dishes were transfected with the pNL4-3 and vesicular stomatitis virus glycoprotein (VSV-G) expression plasmids at a ratio of 4:1 using polyethyleneimine (PolySciences, Warrington, PA). Cell culture supernatants containing infectious virions were collected 2 days posttransfection, filtered, aliquoted, and stored at −80°C. For characterization of the effects of compensatory mutations on virus replication, HEK293T cells were transfected in 24-well plates with pNL4-3-derived plasmids similarly but without VSV-G pseudotyping. Cell lysates collected at 2 days posttransfection were analyzed by immunoblotting as detailed below. In parallel, cell culture supernatants containing virions were titered on TZM-bL cells using a β-galactosidase assay (Galactostar, Thermo Fisher) per manufacturer’s instructions. Virus replication in TZM-bL cells was limited to a single cycle via addition of dextran sulfate (50 μg/mL) 6–14 h postinfection. An aliquot of the viruses was also subjected to a qPCR-based reverse transcriptase (RT) activity assay (95). Virus titers obtained from infection of TZM-bL cells was normalized to particle numbers based on values obtained from RT activity assays to determine the infectiousness (i.e., infectious units/particle) of viruses. A fraction of the cell culture supernatant collected from transfected HEK293T cells was also concentrated by centrifugation (16,100 × *g*, 4°C, 90 min) on a 20% sucrose cushion prepared in 1 × SDS-PAGE sample buffer for analysis of virion-associated proteins by immunoblotting as described below.

### Analysis of compensatory mutations.

Compensatory substitutions that arose in the background of HIV-1_NL4-3 IN (R269A/K273A)_ class II IN mutant virus were isolated by serial passaging as follows. In brief, one million MT4-GFP cells were infected at a multiplicity of infection (MOI) 2 for the WT virus. An equivalent particle number (as determined by a qPCR-based reverse transcriptase (RT) activity assay [95]) of the HIV-1_NL4-3 IN (R269A/K273A)_ virus was used as inoculum. Infected cells were split at a ratio of 1:5 every 3–4 days and infections were monitored by microscopy as well as flow cytometry of GFP-positive cells at the indicated intervals shown in Fig. 1A. Cell culture supernatants containing virions were also collected at the same time intervals for subsequent sequencing analysis. At the end of each passage (i.e., when infections plateaued at >75% GFP+ cells), cell culture supernatants containing infectious virions were collected. The resultant viruses were first titered on MT4-GFP cells and used to infect MT4-GFP cells in the next passage at an MOI of 2 infectious units (i.u.)/cell for WT and an equivalent particle number for the class II IN mutant virus (or its derivative bearing compensatory mutations). Aliquots of infected cells and viral particles in the cell culture supernatants were collected over the duration of each passage as above.

For sequencing analysis of viruses bearing compensatory mutations, virions collected over the three passages were concentrated on 20% sucrose cushions (prepared in 1× phosphate-buffered saline [PBS]) by ultracentrifugation (28,000 rpm, 4°C, 90 min, Beckman SW41 rotor). Genomic RNA was isolated from pelleted virions using TRIzol per manufacturer’s instructions. Extracted RNA was prepared for deep sequencing using the Illumina TruSeq Stranded Total RNA library kit following the manufacturer’s instructions but omitting the rRNA depletion step. Resulting libraries were sequenced on an Illumina HiSeq 2000 platform at the Genome Technology Access Center at Washington University School of Medicine. Sequencing reads were mapped to the pNL4-3 reference viral genome allowing for 2 mismatches using the Bowtie algorithm (i.e., -v 2, -m 10 parameters) (96), and the frequency of compensatory mutations was determined thereafter.

### Immunoblotting.

Viral and cell lysates were resuspended in sodium dodecyl sulfate (SDS) sample buffer, separated by electrophoresis on Bolt 4–12% Bis-Tris Plus gels (Life Technologies) and transferred to nitrocellulose membranes (Hybond ECL, Amersham). The membranes were then probed overnight at 4°C with a mouse monoclonal anti-HIV p24 antibody (183-H12-5C, NIH AIDS reagents) or a mouse monoclonal anti-HIV integrase antibody (97) in Odyssey Blocking Buffer (LI-COR). Membranes were probed with fluorophore-conjugated secondary antibodies (LI-COR) and scanned using a LI-COR Odyssey system. IN and CA levels in virions were quantified using Image Studio software (LI-COR).

### Vpr-IN trans-complementation experiments.

HIV-1_NL4-3_ bearing the class I IN mutation D116N was transcomplemented with class II mutant proteins as previously described (76). In brief, HEK293T cells plated in 24-well dishes were co-transfected with full-length proviral plasmids expressing HIV-1_NL4-3 IN (D116N)_, VSV-G, and derivatives of the pLR2P-VprIN plasmids bearing class II IN mutations (or the compensatory mutations thereof) at a ratio of 6:1:3 using polyethyleneimine as described above. Cell-free virions were collected from cell culture 2 days posttransfection. MT-4 T-cells were infected by the resultant virus stocks, and the integration capability of transcomplemented class II IN mutants was measured by the yield of progeny virions in cell culture supernatants over a 6-day period using the aforementioned reverse transcriptase (RT) activity assay as described before (76). Briefly, MT-4 T-cells were incubated with virus inoculum in 96 V-bottom well plates for 4 h at 37°C before washing away the inoculum and replacing it with fresh media. Right after the addition of fresh media and over the ensuing 6 days, an aliquot of the media was collected and the number of virions in culture supernatant was quantified by measuring a qPCR-based RT activity using assay (95), as also described above.

### CLIP experiments.

CLIP experiments were conducted as previously described (15, 74, 75, 98, 99). In short, cells in 15-cm cell culture plates were transfected with 30 μg full-length proviral plasmid (pNL4-3) DNA or derivatives carrying the IN mutations. 4-thiouridine (4SU) was added to the cell culture media for 16 h before virus harvest. Cell culture supernatants containing virions were filtered through 0.22-μm filters and pelleted by ultracentrifugation through a 20% sucrose cushion prepared in 1× phosphate-buffered saline (PBS) using a Beckman SW32-Ti rotor at 28,000 rpm for 1.5 h at 4°C. The virus pellets were resuspended in 1× PBS and UV-cross-linked for two consecutive times at an energy setting of 500 mJ in a Boekel UV-cross-linking chamber equipped with UV 368-nm bulbs. Following lysis in 1× RIPA buffer, IN-RNA complexes were immunoprecipitated using a mouse monoclonal anti-IN antibody (97). Bound RNA was end-labeled with γ-^32^P-ATP and T4 polynucleotide kinase. The isolated protein-RNA complexes were separated by SDS-PAGE, transferred to nitrocellulose membranes (Hybond ECL, Amersham), and exposed to autoradiography films to visualize IN-RNA complexes. If needed, following immunoblotting and quantitation of immunoprecipitated IN, equivalent amounts of immunoprecipitated IN were rerun to accurately assess the ability of IN variants to bind RNA. Autoradiographs were quantitated by the ImageStudio software (LI-COR). Lysates and immunoprecipitates were also analyzed by immunoblotting using the aforementioned mouse monoclonal antibodies against IN.

### IN multimerization in virions.

HEK293T cells grown in 10-cm dishes were transfected with 10 μg pNL4-3 plasmid DNA bearing WT IN or the indicated *pol* mutations within IN coding sequence as above. Two days posttransfection, cell-free virions in cell culture supernatants were pelleted through a 20% sucrose cushion prepared in 1× PBS using a Beckman SW41-Ti rotor at 28,000 rpm for 1.5 h at 4°C. Pelleted virions were resuspended in 1× PBS and treated with a membrane-permeable cross-linker, EGS (ThermoFisher Scientific), at a concentration of 1 mM for 30 min at room temperature. Crosslinking was stopped by the addition of SDS-PAGE sample buffer. The cross-linked samples were then separated on 3–8% Tris-acetate gels and analyzed by immunoblotting using a mouse monoclonal anti-IN antibody (97).

### Virus production and transmission electron microscopy.

HEK293T cells grown in 15-cm plates were transfected with 30 μg full-length proviral plasmid (pNL4-3) DNA containing WT IN or the indicated *pol* mutations within IN coding sequence as above. Two days posttransfection, cell culture supernatants were filtered through 0.22-μm filters, and pelleted by ultracentrifugation using a Beckman SW32-Ti rotor at 28,000 rpm for 1.5 h at 4°C. Fixative (2% paraformaldehyde/2.5% glutaraldehyde [Polysciences, Inc., Warrington, PA] in 100 mM sodium cacodylate buffer, pH 7.2) was gently added to resulting pellets, and samples were incubated overnight at 4°C. Samples were washed in sodium cacodylate buffer and postfixed in 1% osmium tetroxide (Polysciences, Inc.) for 1 h. Samples were then rinsed extensively in dH_2_0 prior to *en bloc* staining with 1% aqueous uranyl acetate (Ted Pella Inc., Redding, CA) for 1 h. After several rinses in dH_2_0, samples were dehydrated in a graded series of ethanol and embedded in Eponate 12 resin (Ted Pella, Inc.). Sections of 95 nm were cut with a Leica Ultracut UCT ultramicrotome (Leica Microsystems Inc., Bannockburn, IL), stained with uranyl acetate and lead citrate, and viewed on a JEOL 1200 EX transmission electron microscope (JEOL USA Inc., Peabody, MA) equipped with an AMT 8 megapixel digital camera and AMT Image Capture Engine V602 software (Advanced Microscopy Techniques, Woburn, MA). One hundred virions from each sample were classified for virion morphology.

### Quantitation of early and late RT products, 2-LTR circles, and integrated proviral DNA.

We infected 1 × 10^6^ MT4-LTR-GFP indicator cells (per time point/per condition) with equivalent particle numbers of WT and class II IN mutant viruses bearing compensatory mutations. Cells were collected at 4, 8, and 24 hours postinfection (hpi) and pelleted, and total DNA extracted by lysis of cells in Proteinase K (0.1 mg/mL) containing buffer (100 mM NaCl, 10 mM Tris pH 8.0, 25 mM EDTA, 0.5% SDS) for 2 h at 50°C on a thermal mixer set to 1,800 rpm. DNA was extracted by phenol:chloroform:isoamyl alcohol and precipitated with ammonium acetate and ethanol. The resulting DNA pellet was resuspended in 100 μL nuclease-free water, and 5 μL of the sample was subjected to SYBR-green-based qPCR for quantitation of early RT (Forward: 5′-GCTAACTAGGGAACCCACTGCTT; Reverse: 5′-CAACAGACGGGCACACACTACT), late RT (Forward: 5′-TGGGCAAGCAGGGAGCTA; Reverse: 5′-TCCTGTCTGAAGGGATGGTTGT), and 2-LTR (Forward: 5′-CTCAGACCCTTTTAGTCAGTGTGGAAAATCTCTA; Reverse: 5′-TGACCCCTGGCCCTGGTGTGTAG) products as described previously (54). For quantitation of integrated proviral DNA by Alu-PCRs, 4 μL of the DNA samples were subjected to an initial PCR (Forward: 5′-TCCCAGCTACTCGGGAGGCTGAGG; Reverse: 5′-AGGCAAGCTTTATTGAGGCTTAAGC) followed by SYBR green-based qPCR quantification (Forward: 5′-GAAGGGCTAATTCACTCCCA; Reverse: 5′-CTTGAAGTACTCCGGATGCAG) as detailed previously (54).

### Size exclusion chromatography (SEC).

All of the indicated mutations were introduced into a plasmid backbone expressing His_6_ tagged pNL4-3-derived IN by QuikChange site directed mutagenesis kit (Agilent) (67). His_6_ tagged recombinant pNL4-3 WT and mutant IN molecules were expressed in BL21(DE3) E. coli cells followed by nickel and heparin column purification as described previously (67, 100). Recombinant WT and mutant IN molecules were analyzed on Superdex 200 10/300 GL column (GE Healthcare) with running buffer containing 20 mM HEPES (pH 7.5), 1 M NaCl, 10% glycerol, 7.5 mM CHAPS, and 5 mM BME at 0.2 mL/min flow rate. The proteins were diluted to 20 μM with the running buffer and incubated for 1 h at 4°C followed by centrifugation at 10,000 × *g* for 10 min. Multimeric form determination was based on the standards including bovine thyroglobulin (670,000 Da), bovine gamma-globulin (158,000 Da), chicken ovalbumin (44,000 Da), horse myoglobin (17,000 Da), and vitamin B_12_ (1,350 Da). Retention volumes for different oligomeric forms of IN were as follows: tetramer ~12.5 mL, dimer ~14 mL, monomer ~15–16 mL.

### LEDGF/p75 pulldown assay.

The method was modified from that of McKee et al., 2008 (77) in which LEDGF/p75 (2 μM) was incubated with 2 μM His-tagged IN (WT or mutant) in binding buffer (50 mM HEPES (pH 7.5), 200 mM NaCl, 2 mM MgCl_2_, 15 mM imidazole, 0.1% (vol/vol) Nonidet P40) for 60 min at room temperature. Samples were then briefly centrifuged for 2 min at 1,000 × *g*, and supernatants were pulled down by Ni-NTA resin for 30 min in the presence of bovine serum albumin (0.1 mg/mL). The resin was then washed three times with the same buffer, and the bound proteins were separated by SDS-PAGE. LEDGF and INs were separated by SDS–PAGE electrophoresis and visualized by staining with Coomassie-Blue-like AcquaStain (Bulldog-Bio).

### Homogeneous time resolved fluorescence (HTRF).

Catalytic activities of WT and mutant INs were analyzed in the presence of LEDGF/p75 using homogeneous time-resolved fluorescence (HTRF) according to Koneru et al., 2019 (101). Briefly, 100 nM each IN was incubated with 100 nM LEDGF/p75, 50 nM Cy-5 labeled donor DNA, and 10 nM biotinylated target acceptor DNA in 20 mM HEPES (pH 7.5), 1 mM DTT, 10 mM MgCl_2_, 10% glycerol, 0.05% Brij-35, and 0.1 mg/mL BSA. End detection was based on europium-streptavidin that binds to the biotinylated DNA and brings donor europium cryptate closer to acceptor Cy5 fluorophore in integrated DNA. This proximity results in energy transfer to yield a fluorescent signal that was recorded by a PerkinElmer Life Sciences Enspire multimode plate reader. The data were plotted as Ratio, being the ratio of emission signal at 665 nm over that of 615 nm, which was then multiplied by a factor of 10,000.

### Analysis of IN-RNA binding *in vitro*.

To monitor IN-RNA interactions, we utilized AlphaScreen-based assay (38), which allows to monitor the ability of IN to bind and bridge between two TAR RNAs. Briefly, equal concentrations (1 nM) of two synthetic TAR RNA oligonucleotides labeled with either biotin or digoxigenin (DIG) were mixed, and then streptavidin donor and anti-DIG acceptor beads at 0.02 mg/mL concentration were supplied in a buffer containing 100 mM NaCl, 1 mM MgCl_2_, 1 mM DTT, 1 mg/mL BSA, and 25 mM Tris (pH 7.4). After 2 h incubation at 4°C, 320 nM IN was added to the reaction mixture and incubated further for 1.5 h at 4°C. AlphaScreen signals were recorded with a PerkinElmer Life Sciences Enspire multimode plate reader.

### Structural modeling of IN.

Electrostatic potential maps of WT and mutant IN CTDs were created by the Adaptive Poisson-Boltzmann Solver (APBS) program (102) with macromolecular electrostatic calculations performed in PyMOL. The published crystal structure (63) (PDB ID: 5HOT) was used as a template. The calculation results are displayed as an electrostatic potential molecular surface. The low, mid, and high range values are −5, 0, and 5, respectively.

### Analysis of IN mutations from latently infected cells.

Sequences identified from latently infected CD4^+^ T-cells (78) were downloaded from NCBI GenBank based on their accession numbers (KF526120–KF526339). Sequences were imported into the GLUE software framework (103, 104) and aligned. Multiple sequence alignments (MSAs) containing subtype B sequences were constructed using MUSCLE, manually inspected in AliView (105), and imported into a GLUE project database. Within GLUE, MSAs were constrained to the pNL4-3 reference to establish a standardized coordinate space for the gene being analyzed. Amino acid frequencies at each alignment position were summarized using GLUE’s amino-acid frequency calculation algorithm, which accounts for contingencies such as missing data and incomplete codons.
