# Supplementary figures and images for: Emergence of Compensatory Mutations Reveals the Importance of Electrostatic Interactions between HIV-1 Integrase and Genomic RNA
Source: mBio. 2022 Aug 17;13(5):e00431-22. doi: 10.1128/mbio.00431-22 (PMC9601147; doi:10.1128/mbio.00431-22)

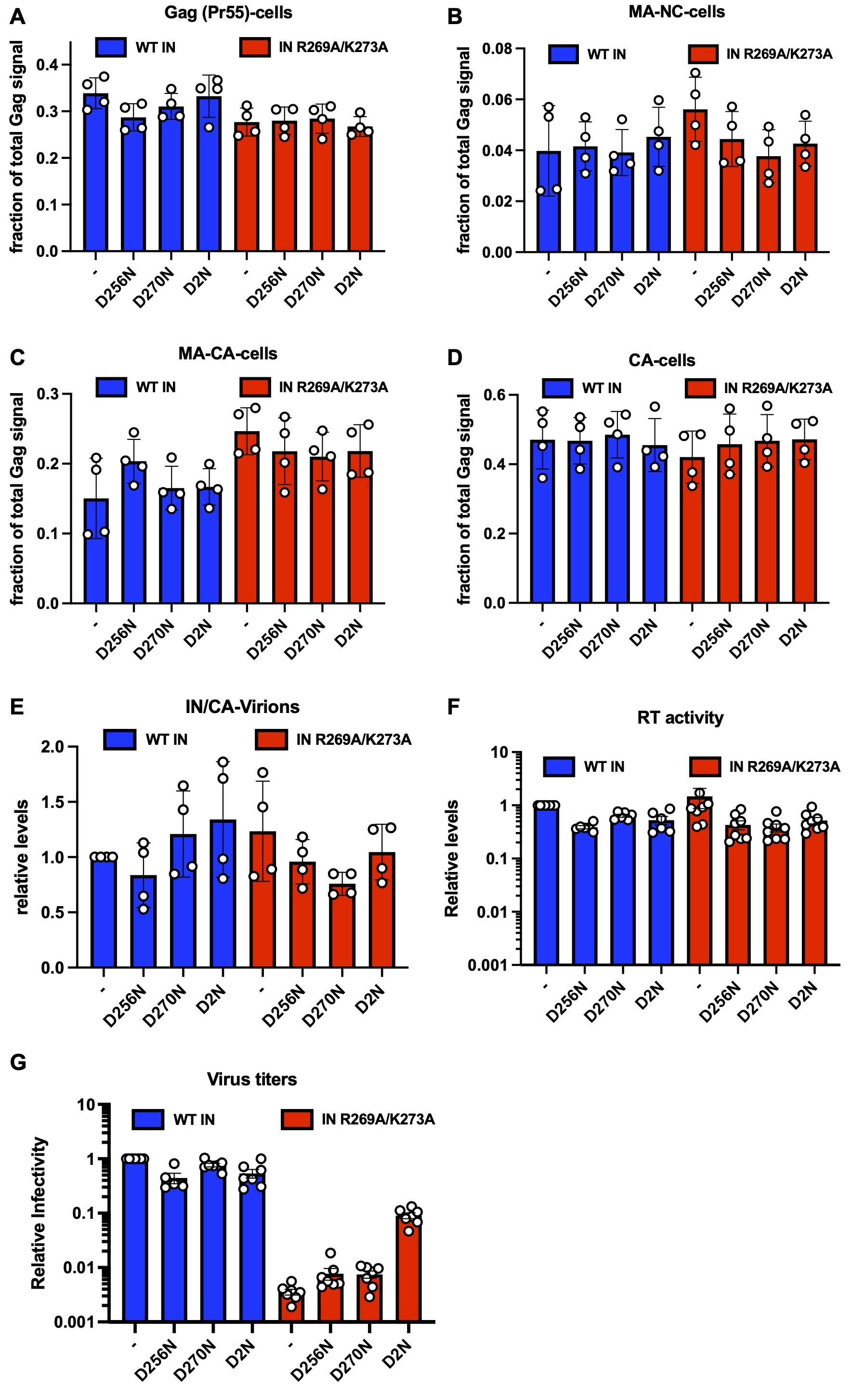

Supplement: FIG S1 [file mbio.00431-22-s0001.tif]

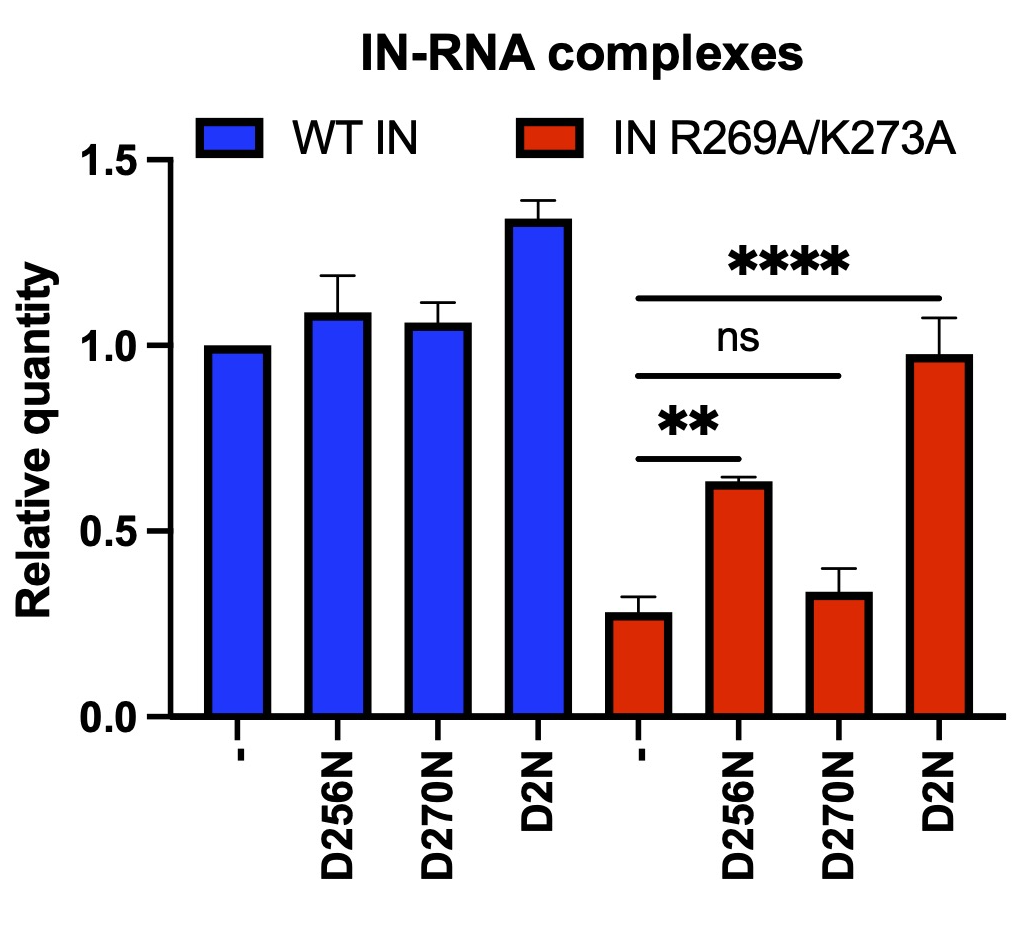

Supplement: FIG S2 [file mbio.00431-22-s0002.tif]

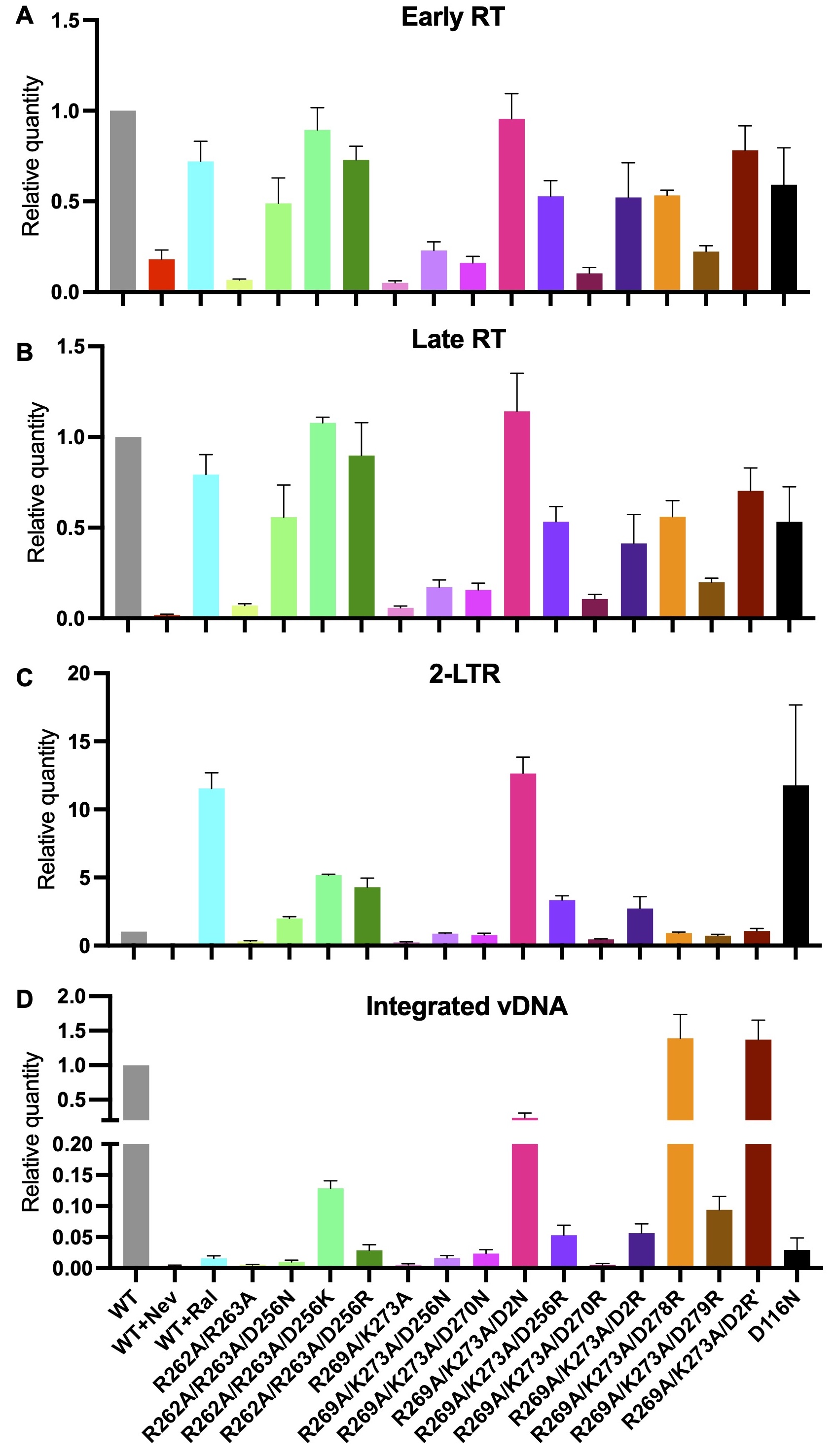

Supplement: FIG S3 [file mbio.00431-22-s0003.tif]

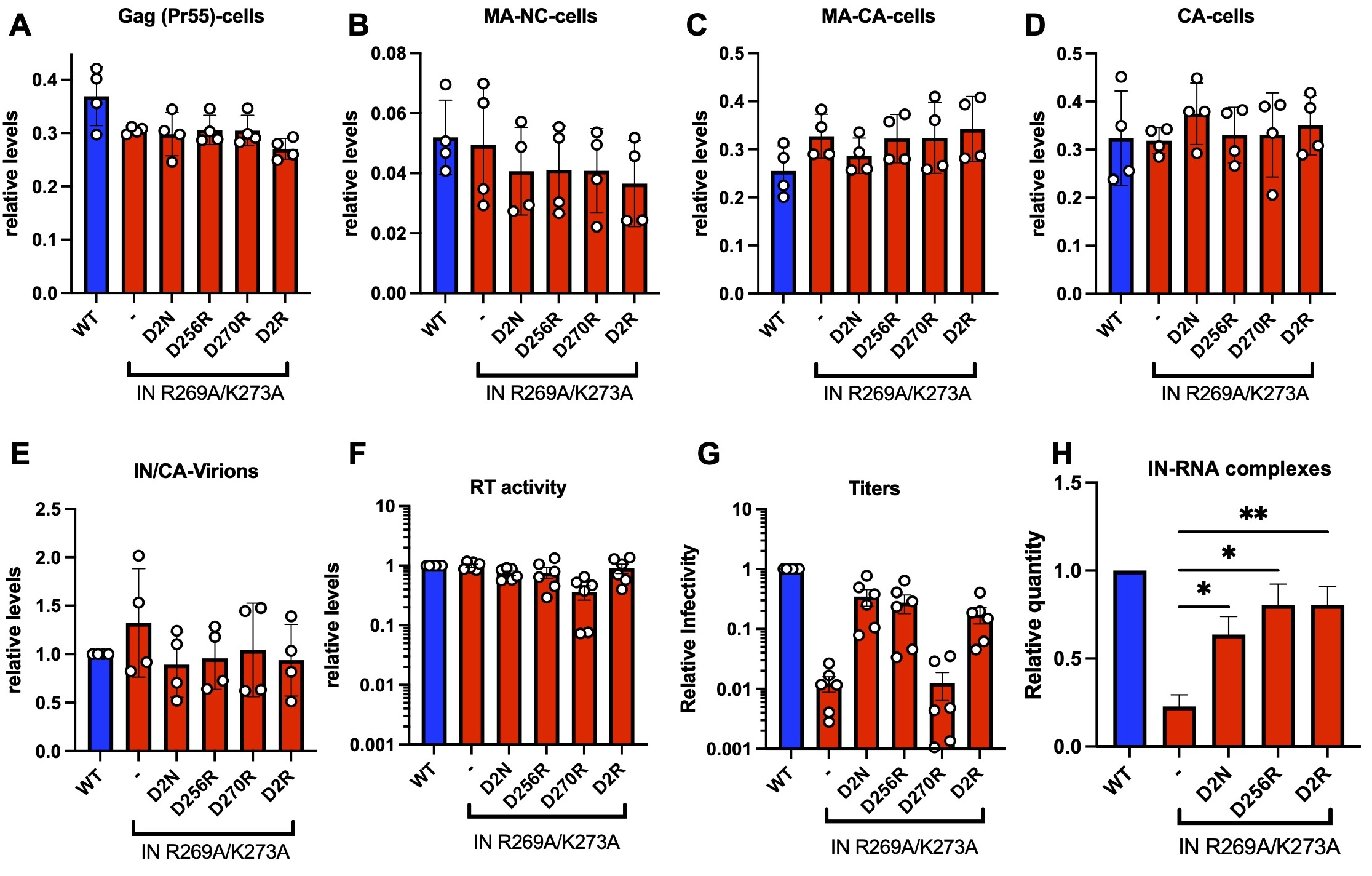

Supplement: FIG S4 [file mbio.00431-22-s0004.tif]

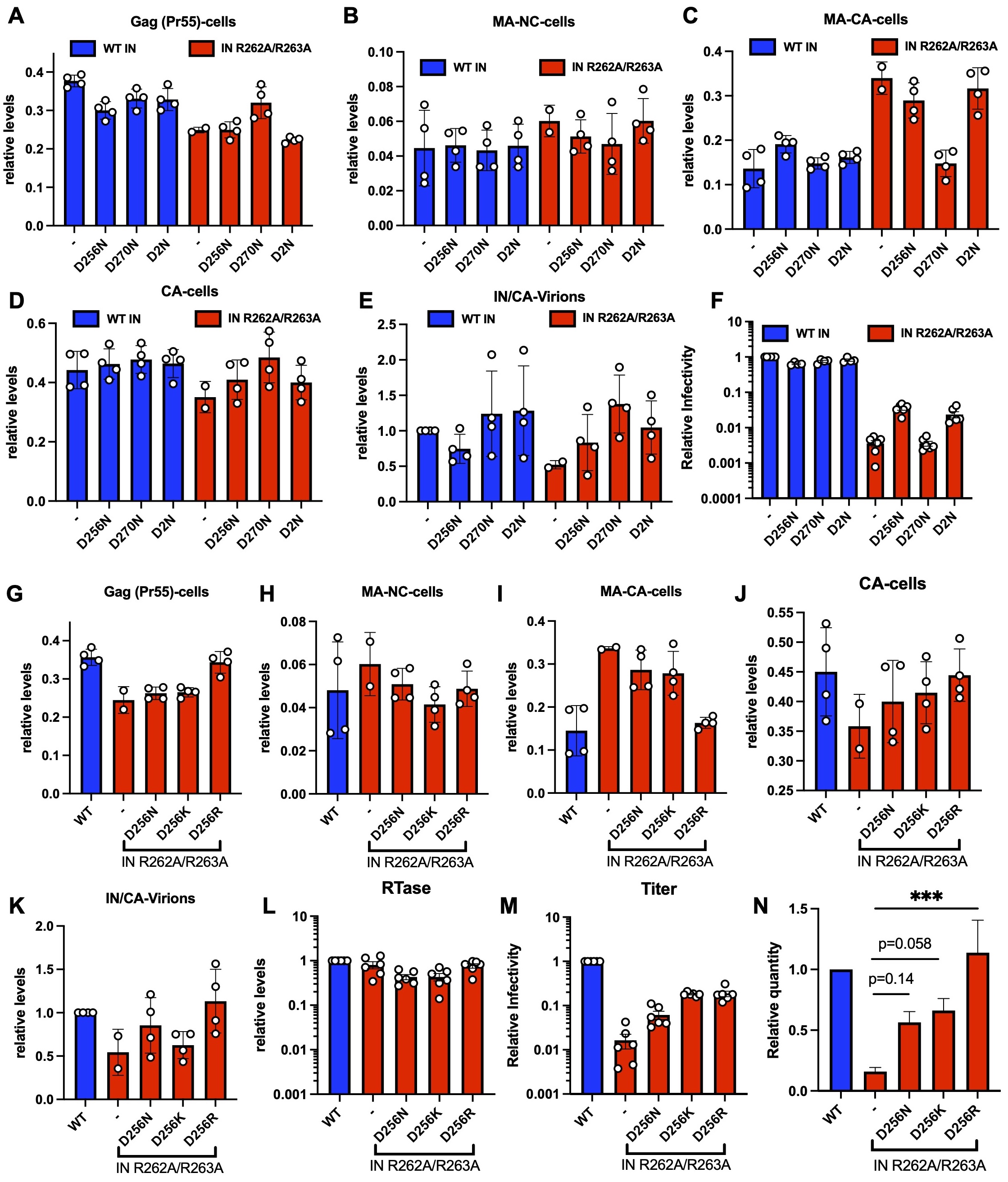

Supplement: FIG S5 [file mbio.00431-22-s0005.jpg]

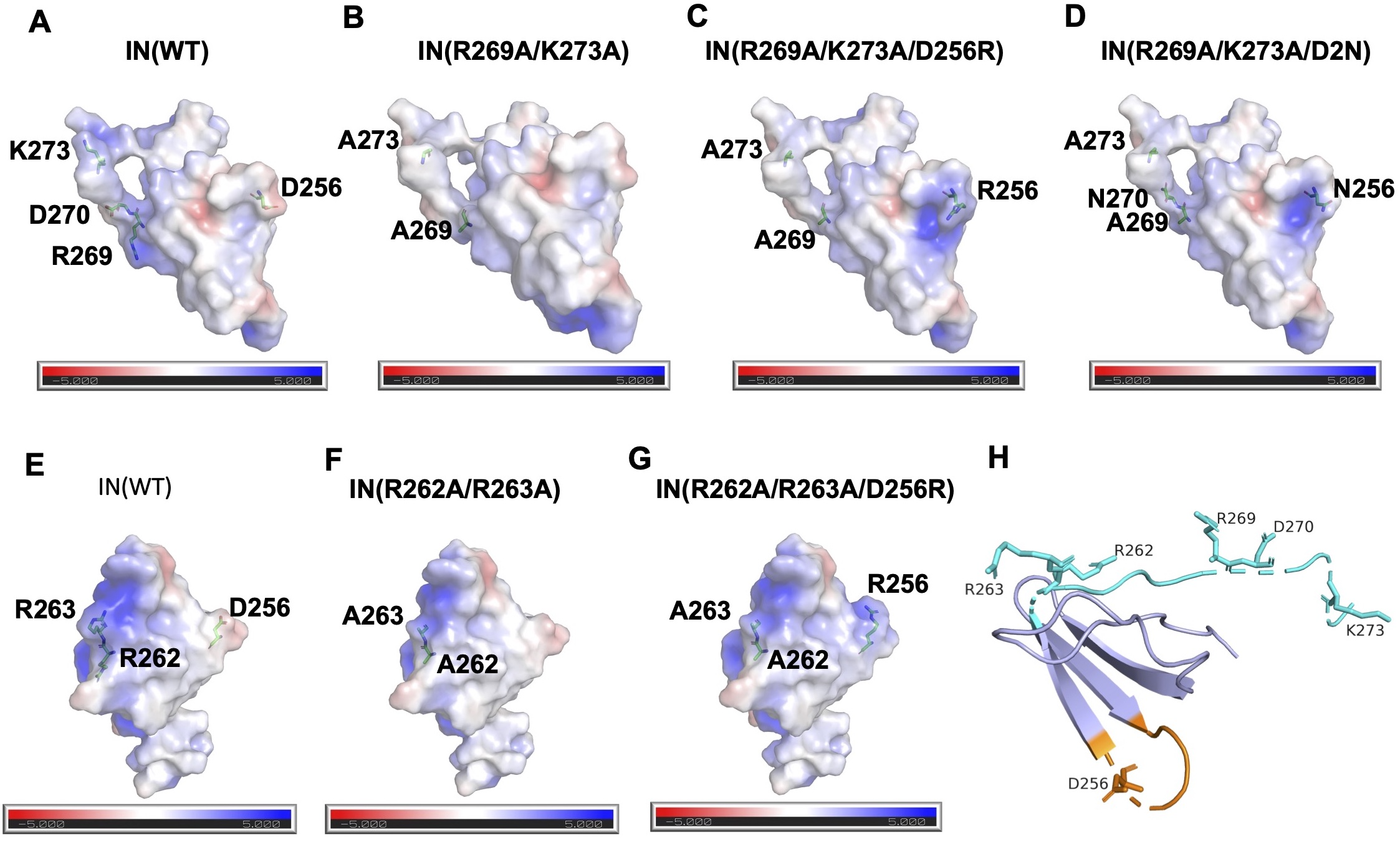

Supplement: FIG S6 [file mbio.00431-22-s0006.tif]
